# Rapid assessment of SARS-CoV-2–evolved variants using virus-like particles

**DOI:** 10.1126/science.abl6184

**Published:** 2021-11-04

**Authors:** Abdullah M. Syed, Taha Y. Taha, Takako Tabata, Irene P. Chen, Alison Ciling, Mir M. Khalid, Bharath Sreekumar, Pei-Yi Chen, Jennifer M. Hayashi, Katarzyna M. Soczek, Melanie Ott, Jennifer A. Doudna

**Affiliations:** 1 Gladstone Institute of Data Science and Biotechnology, San Francisco, CA, USA.; 2 Innovative Genomics Institute, University of California, Berkeley, Berkeley, CA, USA.; 3 Gladstone Institute of Virology, San Francisco, CA, USA.; 4 Biomedical Sciences Graduate Program, University of California, San Francisco, CA, USA.; 5 Department of Molecular and Cell Biology, University of California, Berkeley, CA, USA.; 6 Department of Medicine, University of California San Francisco, CA, USA.; 7 Molecular Biophysics and Integrated Bioimaging Division, Lawrence Berkeley National Laboratory, Berkeley, CA, USA.; 8 Howard Hughes Medical Institute, University of California, Berkeley, Berkeley, CA, USA.; 9 Department of Chemistry, University of California, Berkeley, Berkeley, CA, USA.; 10 California Institute for Quantitative Biosciences (QB3), University of California, Berkeley, Berkeley, CA, USA.; 11 Gladstone-UCSF Institute of Genomic Immunology, San Francisco, CA, USA.

## Abstract

Efforts to determine why new severe acute respiratory syndrome coronavirus 2 (SARS-CoV-2) variants demonstrate improved fitness have been limited to analyzing mutations in the spike (S) protein with the use of S-pseudotyped particles. In this study, we show that SARS-CoV-2 virus-like particles (SC2-VLPs) can package and deliver exogenous transcripts, enabling analysis of mutations within all structural proteins and at multiple steps in the viral life cycle. In SC2-VLPs, four nucleocapsid (N) mutations found universally in more-transmissible variants independently increased messenger RNA delivery and expression ~10-fold, and in a reverse genetics model, the serine-202→arginine (S202R) and arginine-203→methionine (R203M) mutations each produced >50 times as much virus. SC2-VLPs provide a platform for rapid testing of viral variants outside of a biosafety level 3 setting and demonstrate N mutations and particle assembly to be mechanisms that could explain the increased spread of variants, including B.1.617.2 (Delta, which contains the R203M mutation).

## A tool to probe SARS-CoV-2 biology

To develop therapies against severe acute respiratory syndrome coronavirus 2 (SARS-CoV-2) and emerging variants, it is important to understand the viral biology and the effect of mutations. However, this is challenging because live virus can only be studied in a few laboratories that meet stringent safety standards. Syed *et al*. describe a virus-like particle (VLP) that comprises the four SARS-CoV-2 structural proteins, but instead of packaging viral RNA, it packages messenger RNA (mRNA) that expresses a reporter protein (see the Perspective by Johnson and Menachery). The amount of reporter expressed in receiver cells depends on the efficiency of packaging and assembly in the producer cells and the efficiency of entry into receiver cells. Mutations in the nucleocapsid protein that are found in more transmissible variants increase mRNA packaging and expression. The VLPs provide a platform for studying the effect of mutations in the structural proteins and for screening therapeutics. —VV

## Teaser

SARS-CoV-2–like particles enable rapid and safe analysis of viral variants.

The COVID-19 pandemic is a leading cause of death globally, owing to the ongoing emergence of severe acute respiratory syndrome coronavirus 2 (SARS-CoV-2) variants with increased transmissibility. Understanding the molecular determinants of enhanced infectivity is central to vaccine and therapeutic development, but research is hindered because SARS-CoV-2 can be studied only in a biosafety level 3 (BSL-3) laboratory. Furthermore, technical challenges impede efforts to generate mutant infectious clones of SARS-CoV-2 (*1*–*5*). Current studies employ spike (S) protein–pseudotyped lentivirus systems for evaluation of S-mediated ACE2 receptor binding and cell entry (*6*, *7*). However, most mutations in circulating variants occur outside of the S gene and are thus inaccessible by this approach (*8*).

All SARS-CoV-2 variants of interest or concern defined by the World Health Organization contain at least one mutation with >50% penetrance within a seven–amino acid span (199 to 205) in the nucleocapsid (N) protein, which is required for replication, RNA binding, packaging, stabilization, and release (*8*). Despite its functional importance and emergence as a mutational hotspot, the N protein has not been widely studied because of the absence of simple and safe cell-based assays. Biochemical analysis of N has also proven difficult because of its instability and propensity to assemble or phase-separate and to bind RNA nonspecifically (*9*–*11*). To investigate N function, effects of mutations, and other aspects of SARS-CoV-2 biology, we set out to develop a system to package and deliver exogenous RNA transcripts into cells by means of virus-like particles (VLPs).

We reasoned that a process that mimics viral assembly to package and deliver reporter transcripts would simplify the analysis of successful virus production, budding, and entry. Previous studies have shown that coexpression of only the structural proteins of coronaviruses generates VLPs that contain all four structural proteins (*12*–*17*). These VLPs appear to have similar morphology to infectious viruses and have been proposed as vaccine candidates (*18*). A key requirement for such VLPs to deliver reporter transcripts into cells is the recognition of a cis-acting RNA sequence that triggers packaging. During viral assembly, the N protein is thought to recognize one or more RNA structures within open reading frames 1a and 1b (ORF1ab), thus enabling the full viral genome that contains this sequence to be packaged to the exclusion of viral subgenomic and host transcripts (*19*). The identification of such a SARS-CoV-2 cis-acting RNA element is required to create SARS-CoV-2 VLPs (SC2-VLPs) that incorporate and deliver engineered transcripts by this mechanism.

**Fig. 1.**
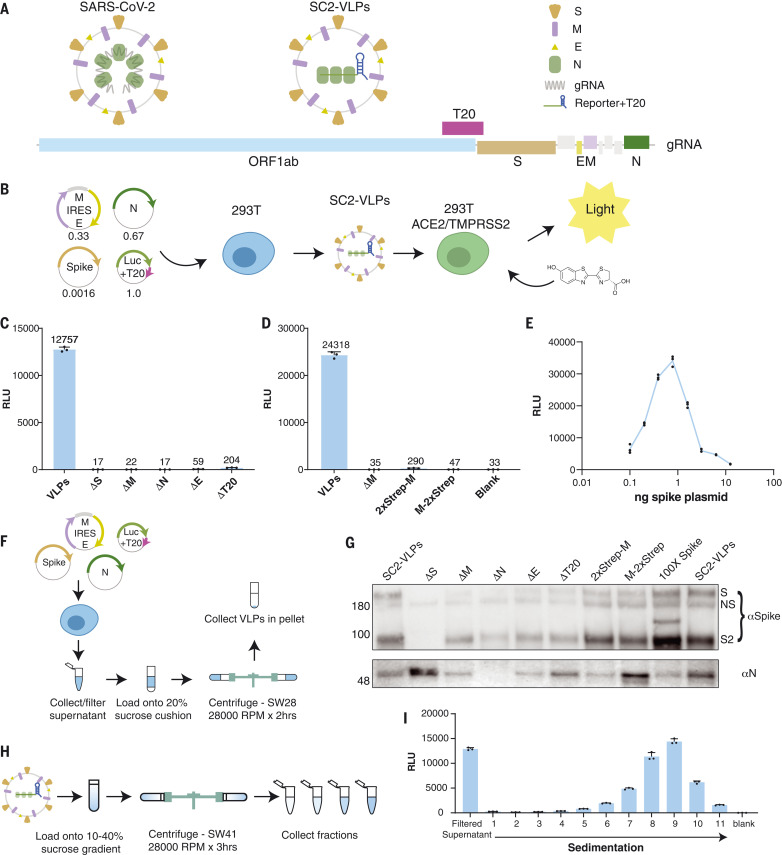
Design and characterization of SC2-VLPs. (**A**) Schematic of SARS-CoV-2 and SC2-VLP design and location of RNA packaging sequence T20. (**B**) Process for generating and detecting luciferase-encoding SC2-VLPs. Numbers below plasmid maps indicate ratios used for transfection. (**C**) Induced luciferase expression measured in receiver cells (293T-ACE2/TMPRSS2) from “standard” SC2-VLPs containing S, M, N, E, and luciferase-T20 transcript, as well as VLPs lacking each one of the components. (**D**) N- or C-terminal 2× Strep-tag on M abrogates vector-induced luciferase expression. (**E**) Optimal luciferase expression requires a narrow range of spike plasmid concentrations corresponding to 1500 of the total plasmid mass used for transfection. (**F**) Schematic for purification of SC2-VLPs. (**G**) Western blot showing S and N in pellets purified from standard SC2-VLPs and conditions that did not induce luciferase expression in receiver cells. (**H**) Schematic for sucrose gradient for separating SC2-VLPs. (**I**) Induced luciferase expression from sucrose gradient fractions of SC2-VLPs. gRNA, genomic RNA; IRES, internal ribosome entry site; Luc, luciferase; RLU, relative light units; S2, cleavage product of S; NS, nonspecific band. Error bars indicate SD with *N* = 3 independent transfections in each case.

On the basis of the reported packaging sequences for related viruses, including murine hepatitis virus and severe acute respiratory syndrome coronavirus (SARS-CoV), we hypothesized that the SARS-CoV-2 packaging signal might reside within a region termed “T20” (nucleotides 20080 to 22222) that encodes nonstructural proteins 15 and 16 (nsp15 and nsp16) (Fig. 1A) (*16*, *19*–*21*). We designed a transfer plasmid that encodes a luciferase transcript containing T20 within its 3′ untranslated region (UTR). We tested for SC2-VLP production by cotransfecting the transfer plasmid into packaging cells (293T cells) along with plasmids that encode the viral structural proteins (Fig. 1B). Supernatant collected from these cells was filtered and incubated with receiver 293T cells that coexpress SARS-CoV-2 entry factors ACE2 and TMPRSS2 (Fig. 1B). We observed luciferase expression in receiver cells only in the presence of all structural proteins (S, M, N, and E) as well as the T20-containing reporter transcript (Fig. 1C). Substituting any one of the structural proteins or the luciferase-T20 transcript with a luciferase-only transcript decreased luminescence in receiver cells >200- and 63-fold, respectively (Fig. 1C). We also conducted this experiment using Vero E6 cells that endogenously express ACE2 and once again observed robust luciferase expression only in the presence of all five components (fig. S1A).

VLP-mediated transcript delivery required untagged native M protein and a low ratio of S expression plasmid relative to the other plasmids (Fig. 1, D and E, and fig. S1B). This is expected because S is expressed at lower levels than other structural proteins during infection (*22*). SC2-VLPs should also require lower S expression compared with pseudovirus systems because S assembles along with the other structural proteins within the endoplasmic reticulum–Golgi intermediate compartment, whereas pseudovirus assays typically require accumulation of S at the plasma membrane followed by random incorporation into budding particles (*23*, *24*). Notably, we detected N and S proteins within pelleted material (Fig. 1, F and G) from multiple conditions that did not yield luciferase expression in receiver cells, suggesting that particles produced under less stringent conditions are not competent for delivering mRNA. These findings suggest that coexpression of structural proteins likely produces VLPs as well as defective particles, and mRNA delivery requires more stringent conditions.

Further analysis showed that SC2-VLPs are stable against ribonuclease A; are resistant to freeze-thaw treatment (fig. S2A); can be concentrated by precipitation, ultrafiltration, and ultracentrifugation (fig. S2B); and induce transient expression of luciferase (fig. S2C). Analysis of SC2-VLPs by sucrose gradient ultracentrifugation showed that large, dense particles are responsible for inducing luciferase expression (Fig. 1, H and I). These data show that SC2-VLPs are formed under our experimental conditions and deliver selectively packaged transcripts.

**Fig. 2.**
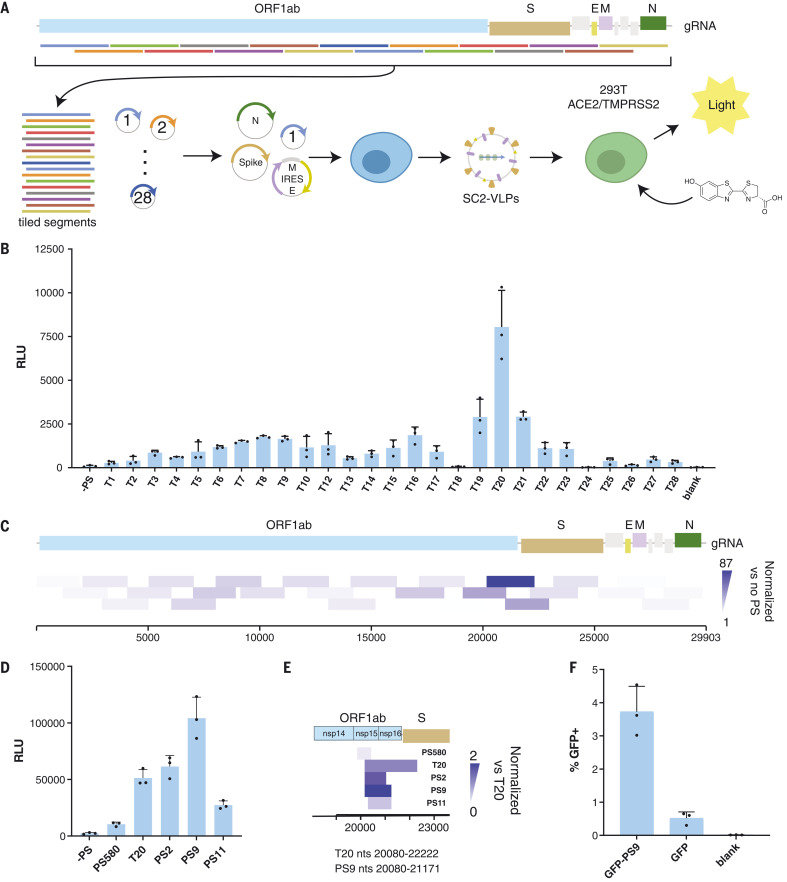
RNA packaging into SC2-VLPs by SARS-CoV-2 sequences. (**A**) Arrayed screen for determining the location of the optimal sequence for RNA packaging in SC2-VLPs. Two-kilobase tiled segments of the genome were cloned into the 3′ UTR of the luciferase plasmid. (**B**) Induced luciferase expression in receiver cells by SC2-VLPs containing different tiled segments from the SARS-CoV-2 genome. (**C**) Heatmap visualization of the data from (B), showing the locations of tiled segments relative to the SARS-CoV-2 genome. Color intensity indicates luminescence of receiver cells for each tile normalized to expression for a luciferase plasmid containing no insert. (**D**) Smaller segments of the genome were used to locate the optimal RNA packaging sequence. (**E**) Heatmap visualization of the data from (D). (**F**) Flow cytometry analysis of GFP expression in 293T ACE2/TMPRSS2 cells incubated with SC2-VLPs encoding GFP-PS9, GFP (no packaging sequence), or no VLPs. nts, nucleotides. Error bars indicate SD with *N* = 3 independent transfections in each case.

Next, we determined the optimal SARS-CoV-2 cis-acting RNA sequence for SC2-VLP–mediated delivery. We generated a library of 28 overlapping tiled segments of 2 kb each (numbered T1 to T28; table S1) from the SARS-CoV-2 genome and inserted them individually into a luciferase-encoding plasmid (Fig. 2A). SC2-VLPs generated using luciferase-encoding plasmids that included any region of ORF1ab produced detectable luminescence, suggesting that SARS-CoV-2 packaging may not rely entirely on one contiguous cis-acting RNA element (Fig. 2, B and C). Furthermore, luciferase-encoding plasmids that included fragments T24 to T28 resulted in lower levels of luciferase expression (Fig. 2, B and C), consistent with the exclusion of subgenomic viral transcripts containing these sequences to minimize production of replication-defective virus particles. Overall, luciferase expression was most efficient using T20 (nucleotides 20080 to 22222) located near the 3′ end of ORF1ab (Fig. 2, B and C) and partially but not completely overlapping with PS580 (nucleotides 19785 to 20348), which was previously predicted to be the packaging signal for SARS-CoV on the basis of structural similarity to known coronavirus packaging signals (*16*).

To further define the minimal cis-acting RNA element sufficient for SC2-VLP–mediated delivery, we tested truncations and additions to T20 as well as PS580 from SARS-CoV. We found that PS580 resulted in lower luciferase expression compared with T20 (Fig. 2, D and E, and fig. S3, A and B). We observed the highest luciferase expression level from SC2-VLPs encoding the nucleotide sequence 20080 to 21171 (termed PS9), and further truncations of this sequence reduced expression (Fig. 2, D and E, and fig. S3, A and B). We used PS9 to generate VLPs encoding green fluorescent protein (GFP) and found that they induced GFP expression in receiver cells (Fig. 2F). These data suggest that PS9 (nucleotides 20080 to 21171) is a cis-acting element that is sufficient for triggering RNA packaging into SC2-VLPs, although it is not currently known whether this sequence is required for the packaging of the SARS-CoV-2 genome.

**Fig. 3.**
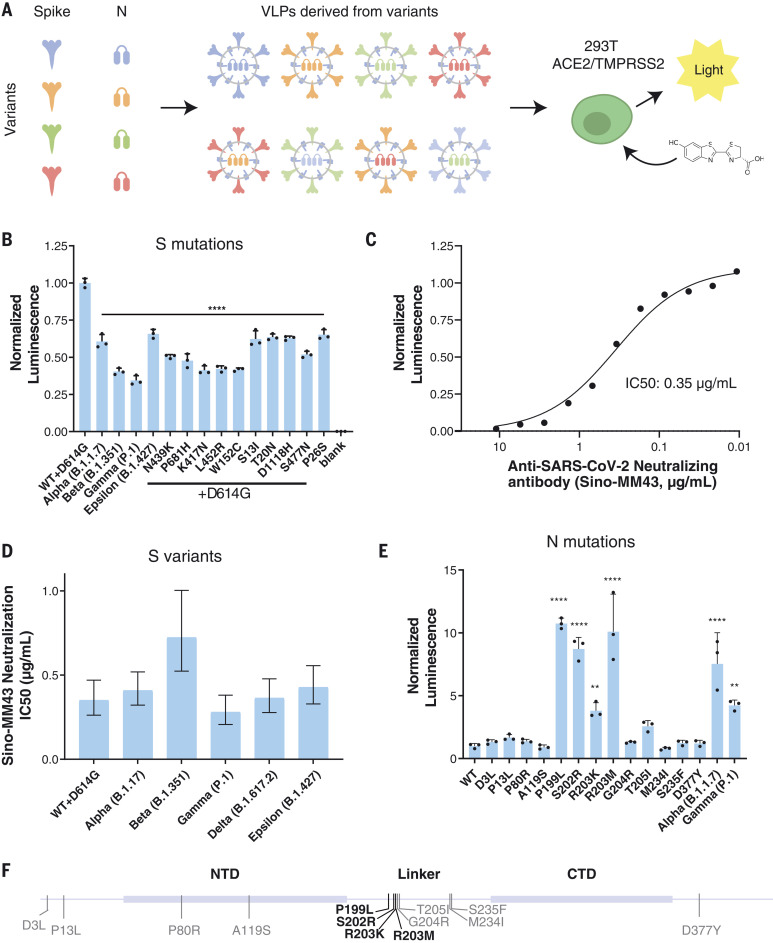
Effect of mutations in the S and N proteins on SC2-VLP–induced luminescence. (**A**) Schematic for cloning and testing mutations observed in SARS-CoV-2 variants using SC2-VLPs. (**B**) Initial screen of 15 S mutants compared with a reference ancestral S containing the D614G mutation (termed WT). Details of mutations are listed in table S2. (**C**) Neutralization curve for SC2-VLPs generated using ancestral S and neutralized with the anti-S antibody MM43 (SinoBiological, catalog no. 40591). (**D**) Neutralization IC_50_ of S variants using SC2-VLPs and MM43. (**E**) Initial screen of 15 N mutants compared with the reference Wuhan Hu-1 N sequence (WT). Details of mutations are listed in table S3. (**F**) Map of SARS-CoV-2 N domains showing the locations of observed mutations. Mutations that were observed to enhance signal are shown in bold. (B and E) Error bars indicate SD with *N* = 3 independent transfections in each case. Significance was determined by one-way analysis of variance and multiple comparisons using the Holm-Šídák test. **P < 0.01; ****P < 0.0001. (D) Error bars indicate 95% confidence intervals derived from curve fitting in GraphPad Prism. NTD, N-terminal domain; CTD, C-terminal domain. Single-letter abbreviations for the amino acid residues are as follows: A, Ala; C, Cys; D, Asp; F, Phe; G, Gly; H, His; I, Ile; K, Lys; L, Leu; M, Met; N, Asn; P, Pro; R, Arg; S, Ser; T, Thr; W, Trp; and Y, Tyr.

Compared with pseudoviruses, SC2-VLPs provide a new and more physiological model for testing mutations in all viral structural proteins (S, E, M, and N) for effects on assembly, packaging, and cell entry. Surprisingly, none of the 15 SC2-VLPs generated with S mutant genes—including four with the combined mutations found in the B.1.1.7 (Alpha), B.1.351 (Beta), P.1 (Gamma), and B.1.427 (Epsilon) variants—increased luciferase expression in transduced cells beyond that observed for the original SC2-VLPs (Fig. 3, A and B, and table S2). Because nearly all circulating variants contain the S D614→G (S:D614G) mutation, we compared all mutants to the ancestral S protein modified to include G614 (termed WT+D614G). Minor changes in S expression between mutants may be a confounding factor because SC2-VLPs mediate luciferase expression optimally in a narrow range of S expression. Over a range of 6.25 ng to 50 pg per well of S-encoding plasmid, none of the tested S mutations produced >twofold improvement in luciferase expression (fig. S4, A and B); slightly improved luciferase expression occurred with the S sequence derived from the Alpha variant (B.1.1.7) and with S containing the mutation N501Y within the receptor binding domain. These findings are in contrast to prior results for S-pseudotyped lentiviruses, for which enhanced entry was reported for some S mutations, including S:N501Y (*25*, *26*). However, S mutations tested in the context of SARS-CoV-2 infectious clones have shown mixed effects, and mutations within S may also mediate enhanced transmission by interfering with the binding of neutralizing antibodies (*27*, *28*). We tested whether VLPs could also be used to measure antibody neutralization and found results similar to previously reported data (Fig. 3C and fig. S4C). Using a neutralizing monoclonal antibody (MM43), we observed dose-dependent inhibition of luminescence with a measured median inhibitory concentration (IC_50_) of 0.35 μg/ml, similar to the manufacturer-reported IC_50_ of 1.41 μg/ml. We also tested S from circulating variants and observed robust MM43-mediated neutralization of SC2-VLPs generated from Alpha, Beta, Gamma, B.1.617.2 (Delta), and Epsilon variant S proteins (Fig. 3D) and consistent with previous studies (*29*). These results show that SC2-VLPs employ S-mediated entry and can be used for screening S mutations for entry and neutralization. Although we did not observe enhanced entry resulting from the S mutations we tested, these mutations could still provide a fitness advantage for SARS-CoV-2 by limiting antibody-mediated neutralization. Detailed characterization of S mutations and their sensitivity to neutralizing antibodies has been examined in other studies (*30*, *31*).

We next used SC2-VLPs to test whether N mutations found in circulating variants improve viral particle assembly, RNA delivery, and/or reporter gene expression. We tested 15 N mutations, including two combinations corresponding to the Alpha and Gamma variants because they both contain the co-occurring R203K and G204R mutations. Alpha and Gamma variant N proteins improved luciferase expression in receiver cells by 7.5- and 4.2-fold, respectively, relative to the ancestral Wuhan Hu-1 N protein (Fig. 3E). In addition, four single–amino acid changes—P199L, S202R, R203K, and R203M—improved luciferase expression. Two of these mutations (P199L and R203K) do not change the overall charge, one (S202R) results in a more positive charge, and one (R203M) results in a more negative charge, which suggests that the improvement in luciferase expression is not likely due to simple electrostatics. Western blotting revealed no correlation between N protein expression levels and luciferase induction, suggesting that these N mutations enhance luciferase induction through a different mechanism (fig. S5). Notably, half of the amino acid changes observed within N and all of the mutations observed to enhance luminescence occur within a seven–amino acid span (199 to 205) of the central disordered region (termed the “linker” region; Fig. 3F), suggestive of a shared mechanism.

**Fig. 4.**
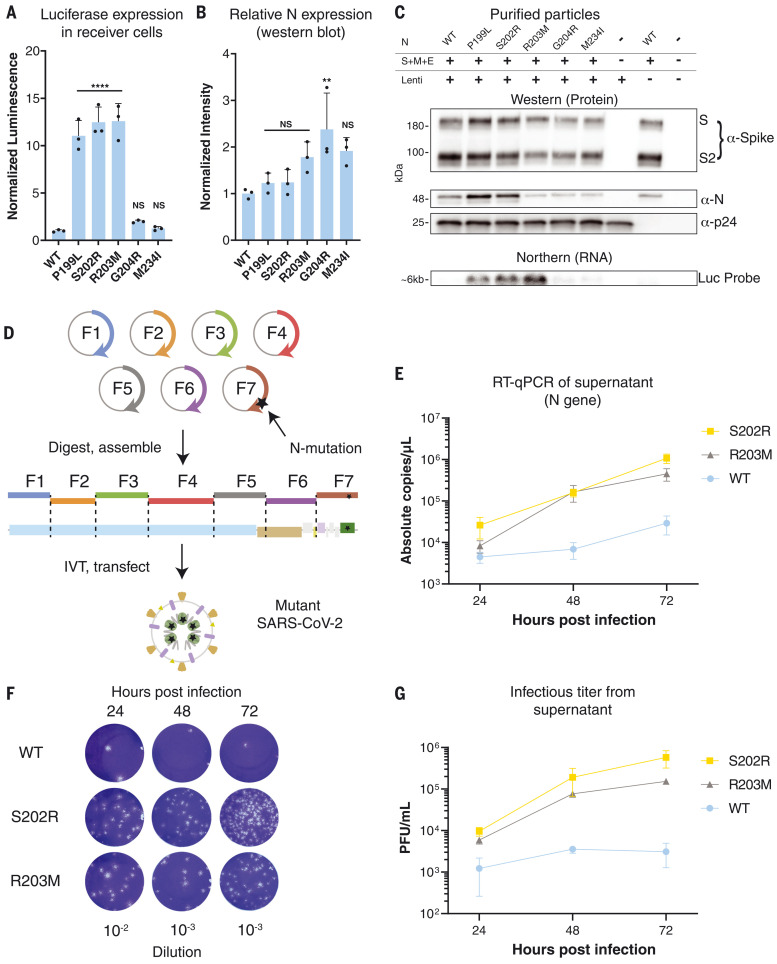
Impact of mutations in SARS-CoV-2 N on RNA packaging and viral titer. (**A**) Luciferase expression in receiver cells from six N mutants retested after preparation in a larger batch. (**B**) Relative N-expression of selected mutants in packaging cells normalized to WT, with glyceraldehyde-3-phosphate dehydrogenase as a loading control. (**C**) Western blot (protein) and Northern blot (RNA) of VLPs generated using N-mutants purified by ultracentrifugation. Lentivirus was added before ultracentrifugation to allow use of p24 as an internal control. (**D**) Schematic for the reverse genetics system used to generate mutant SARS-CoV-2. (**E**) RT-qPCR of supernatant collected from A549-ACE2 cells infected with WT and mutant SARS-CoV-2 at MOI of 0.1 at 24, 48, and 72 hours after infection. (**F**) Representative plaques and (**G**) quantification of infectious viral titers from the same experiment. Error bars indicate SD with *N* = 3 independent transfections or infections in each case. Significance was determined by one-way analysis of variance and multiple comparisons using Holm-Šídák test. **P < 0.01; ****P < 0.0001; NS, not significant. F1 to F7, fragments 1 to 7; IVT, in vitro transcription; PFU, plaque-forming units.

Further analysis of six N mutants was conducted to determine whether these mutations affect SC2-VLP assembly efficiency, RNA packaging, or RNA uncoating prior to expression. We chose the three mutants for which luciferase expression was improved by a factor of ~10 (P199L, S202R, and R203M) and two mutants that did not result in significantly increased luciferase expression (G204R and M234I) in the preliminary screen and repeated the measurement of their induced luciferase expression (Fig. 4A). N protein expression levels were similar in packaging cells, except for G204R, and did not correlate with luciferase expression (Fig. 4, A and B). We then purified SC2-VLPs containing each N mutation and found that those containing P199L and S202R had increased levels of S, N, and luciferase RNA, whereas R203M showed increased luciferase RNA only (Fig. 4C). These results suggest that mutations within the N linker domain improve the assembly of SC2-VLPs, leading to greater overall VLP production, a larger fraction of VLPs that contain RNA, or higher RNA content per particle. In each case, these results suggest a previously unanticipated explanation for the increased fitness and spread of SARS-CoV-2 variants of concern.

To validate whether the effects we observed in SC2-VLPs improve replication of intact virus, we used reverse genetics to generate SARS-CoV-2 containing N:S202R and N:R203M substitutions within a USA/WA1-2020 (Washington isolate) background (*1*) (Fig. 4D). We generated and used next-generation sequencing to verify stocks of virus containing the indicated mutations (fig. S6). We infected A549-ACE2 cells with wild type, N:S202R, or N:R203M at a multiplicity of infection (MOI) of 0.1 and collected supernatants at 24, 48, and 72 hours after infection. Reverse transcription quantitative polymerase chain reaction (RT-qPCR) indicated 45 and 23 times the RNA content in the supernatant at 72 hours after infection, and plaque assays indicated 166 and 51 times the infectious titers for N:S202R and N:R203M virus, respectively (Fig. 4, E to G). Our results indicate that both of these mutations enhance replication in lung epithelial cells, consistent with our observations using SC2-VLPs.

Overall, we present a strategy for rapidly generating and analyzing SC2-VLPs that package and deliver exogenous mRNA. This approach allows examination of viral assembly, budding, stability, maturation, entry, and genome uncoating involving all of the viral structural proteins (S, E, M, and N) without generating replication-competent virus. Such a strategy is useful not only for dissecting the molecular virology of SARS-CoV-2 but also for future development and screening of therapeutics to target assembly, budding, maturation, and entry. This strategy is ideally suited for the development of new antivirals targeting SARS-CoV-2, as it is sensitive, quantitative, and scalable to high-throughput workflows. The unexpected finding of improved mRNA packaging and luciferase induction by mutations within the N protein points to a previously unknown strategy for coronaviruses to evolve enhanced viral fitness. The mechanism for this enhancement involves increased mRNA packaging and delivery, although the exact process is currently unknown. Recent literature suggests that these mutations may affect phosphorylation of N, its binding to RNA, and phase separation behavior—all of which could affect the efficiency of assembly (*32*–*34*). Our observations of enhanced RNA packaging and replication are consistent with recent reports that the Delta variant (containing N:R203M) generates viral RNA levels elevated by a factor of 1000 within patients (*35*). Our results provide a molecular basis to explain why the SARS-CoV-2 Delta variant demonstrates improved viral fitness.

## Supplementary Material

20211104-1

## Supplementary Materials

### This PDF file includes:

Materials and Methods Figs. S1 to S6 Tables S1 to S3 References

### Other Supplementary Material for this manuscript includes the following:

MDAR Reproducibility Checklist

## Notes

View/request a protocol for this paper from *Bio-protocol*.
