# Supplementary material for: Rapid assessment of SARS-CoV-2–evolved variants using virus-like particles
Source: Science. 2021 Nov 4;374(6575):1626–32. doi: 10.1126/science.abl6184 (PMC9005165; doi:10.1126/science.abl6184)
Supplement: 20211104-1 [file science.abl6184.v1.pdf]

Cite as: A. M. Syed *et al.*, *Science*
10.1126/science.abl6184 (2021).

Rapid assessment of SARS-CoV-2 evolved variants using virus-like particles

Abdullah M. Syed[1,2]†, Taha Y. Taha[3]†, Takako Tabata[3]†, Irene P. Chen[3,4], Alison Ciling[2], Mir M. Khalid[3], Bharath Sreekumar[3], Pei-Yi Chen[3], Jennifer M. Hayashi[3], Katarzyna M. Soczek[2,5], Melanie Ott[2,3,6]*, Jennifer A. Doudna[1,2,5,7,8,9,10]*

[1]Gladstone Institute of Data Science and Biotechnology, San Francisco, CA, USA. [2]Innovative Genomics Institute, University of California, Berkeley, Berkeley, CA, USA. [3]Gladstone Institute of Virology, San Francisco, CA, USA. [4]Biomedical Sciences Graduate Program, University of California, San Francisco, CA, USA. [5]Department of Molecular and Cell Biology, University of California, Berkeley, CA, USA. [6]Department of Medicine, University of California San Francisco, CA, USA. [7]Molecular Biophysics and Integrated Bioimaging Division, Lawrence Berkeley National Laboratory, Berkeley, CA, USA. [8]Howard Hughes Medical Institute, University of California, Berkeley, Berkeley, CA, USA. [9]Department of Chemistry, University of California, Berkeley, Berkeley, CA, USA. [10]California Institute for Quantitative Biosciences (QB3), University of California, Berkeley, Berkeley, CA, USA.

†These authors contributed equally to this work.

***Corresponding author. Email: doudna@berkeley.edu (J.A.D.); melanie.ott@gladstone.ucsf.edu (M.O.)**

Efforts to determine why new SARS-CoV-2 variants demonstrate improved fitness have been limited to analyzing mutations in the spike (S) protein using S-pseudotyped particles. Here we show that SARS-CoV-2 virus-like particles (SC2-VLPs) can package and deliver exogenous transcripts, enabling analysis of mutations within all structural proteins and at multiple steps in the viral life cycle. In SC2-VLPs, four nucleocapsid (N) mutations found universally in more-transmissible variants independently increased mRNA delivery and expression by ~10-fold, and in a reverse genetics model, S202R and R203M each produced >50-fold more virus. SC2-VLPs provide a platform for rapid testing of viral variants outside a biosafety level 3 setting and demonstrate N mutations and particle assembly to be mechanisms that could explain the increased spread of variants, including Delta (R203M).

The COVID-19 pandemic is a leading cause of death globally due to the ongoing emergence of SARS-CoV-2 variants with increased transmissibility. Understanding the molecular determinants of enhanced infectivity is central to vaccine and therapeutic development, but research is hindered because SARS-CoV-2 can only be studied in a biosafety level 3 (BSL-3) lab. Furthermore, generating mutant infectious clones of SARS-CoV-2 is technically challenging (*1*–*5*). Current studies employ spike (S) pseudotyped lentivirus systems for evaluation of S-mediated ACE2 receptor binding and cell entry (*6*, *7*). However, most mutations in circulating variants occur outside the S gene and are thus inaccessible by this approach (*8*).

All SARS-CoV-2 variants of interest or concern defined by the WHO contain at least one mutation with >50% penetrance within seven amino acids (N:199-205) in the nucleocapsid (N) protein, which is required for replication and RNA binding, packaging, stabilization and release (*8*). Despite its functional importance and emergence as a mutational hotspot, the N protein has not been widely studied because of the absence of simple and safe cell-based assays. Biochemical analysis of N has also proven difficult because of its instability and propensity to assemble or phase-separate and to bind RNA non-specifically (*9*–*11*). To investigate N function, effects of mutations and other aspects of SARS-CoV-2 biology, we set out to develop a system to package and deliver exogenous RNA transcripts into cells using virus-like particles (VLPs).

We reasoned that a process mimicking viral assembly to package and deliver reporter transcripts would simplify the analysis of successful virus production, budding and entry. Previous studies have shown that co-expression of only the structural proteins of coronaviruses generates VLPs containing all four structural proteins (*12*–*17*). These VLPs appear to have similar morphology to infectious viruses and have been proposed as vaccine candidates (*18*). A key requirement for such VLPs to deliver reporter transcripts into cells is the recognition of a cis-acting RNA sequence that triggers packaging. During viral assembly, the N protein is thought to recognize one or more RNA structures within ORF1ab, enabling the full viral genome that contains this sequence to be packaged to the exclusion of viral subgenomic and host transcripts (*19*). The identification of such a SARS-CoV-2 cis-acting RNA element is required to create SARS-CoV-2 VLPs (SC2-VLPs) that incorporate and deliver engineered transcripts by this mechanism.

Based on the reported packaging sequences for related viruses, including Murine Hepatitis Virus and SARS-CoV-1, we hypothesized that the SARS-CoV-2 packaging signal might reside within a region we termed “T20” (nt 20080-22222)

encoding non-structural protein 15 (nsp15) and nsp16 (Fig. 1A) (*16*, *19*–*21*). We designed a transfer plasmid encoding a luciferase transcript containing T20 within its 3′ untranslated region (UTR). We tested for SC2-VLP production by co-transfecting the transfer plasmid into packaging cells (293T) along with plasmids encoding the viral structural proteins (Fig. 1B). Supernatant collected from these cells was filtered and incubated with receiver 293T cells co-expressing SARS-CoV-2 entry factors ACE2 and TMPRSS2 (Fig. 1B). We observed luciferase expression in receiver cells only in the presence of all structural proteins (S, M, N, E) as well as the T20-containing reporter transcript (Fig. 1C). Substituting any one of the structural proteins or the luciferase-T20 transcript with a luciferase-only transcript decreased luminescence in receiver cells by >200-fold and 63-fold, respectively (Fig. 1C). We also conducted this experiment using Vero E6 cells that endogenously express ACE2 and once again observed robust luciferase expression only in the presence of all five components (fig. S1A).

VLP-mediated transcript delivery required untagged native M protein and a low ratio of S expression plasmid relative to the other plasmids (Fig. 1, D and E, and fig. S1B). This is expected since S is expressed at lower levels compared to other structural proteins during infection (*22*). SC2-VLPs should also require lower S expression compared to pseudovirus systems because S assembles along with the other structural proteins within the ER-Golgi intermediate compartment, while pseudovirus assays typically require accumulation of S at the plasma membrane followed by random incorporation into budding particles (*23*, *24*). Interestingly, we detected N and S proteins within pelleted material (Fig. 1, F and G) from multiple conditions that did not yield luciferase expression in receiver cells, suggesting that particles produced under less stringent conditions are not competent for delivering mRNA. These findings suggest that co-expression of structural proteins likely produces VLPs as well as defective particles, and mRNA delivery requires more stringent conditions.

Further analysis showed that SC2-VLPs are stable against ribonuclease A, resistant to freeze-thaw treatment (fig. S2A), can be concentrated by precipitation, ultrafiltration and ultracentrifugation (fig. S2B) and induce transient expression of luciferase (fig. S2C). Analysis of SC2-VLPs by sucrose gradient ultracentrifugation showed that large dense particles are responsible for inducing luciferase expression (Fig. 1, H and I). These data show that SC2-VLPs are formed under our experimental conditions and deliver selectively packaged transcripts.

Next, we determined the optimal SARS-CoV-2 cis-acting RNA sequence for SC2-VLP mediated delivery. We generated a library of 28 2-kb overlapping tiled segments (numbered T1-T28, table S1) from the SARS-CoV-2 genome and inserted them individually into a luciferase-encoding plasmid (Fig. 2A). SC2-VLPs generated using luciferase-encoding plasmids that included any region of ORF1ab produced detectable luminescence, suggesting that SARS-CoV-2 packaging may not rely entirely on one contiguous cis-acting RNA element (Fig. 2, B and C). Furthermore, luciferase-encoding plasmids that included fragments T24-28 resulted in lower luciferase expression (Fig. 2, B and C), consistent with the exclusion of subgenomic viral transcripts containing these sequences to minimize production of replication-defective virus particles. Overall, luciferase expression was most efficient using T20 (nt 20080 - 22222) located near the 3′ end of ORF1ab (Fig. 2, B and C) and partially but not completely overlapping with PS580 (19785-20348), which was previously predicted to be the packaging signal for SARS-CoV-1 based on structural similarity to known coronavirus packaging signals (*16*).

To further define the minimal cis-acting RNA element sufficient for SC2-VLP mediated delivery, we tested truncations and additions to T20 as well as PS580 from SARS-CoV-1. We found that PS580 resulted in lower luciferase expression compared to T20 (Fig. 2, D and E, and fig. S3, A and B). We observed the highest luciferase expression level from SC2-VLPs encoding the nucleotide sequence 20080-21171 (termed PS9), and further truncations of this sequence reduced expression (Fig. 2, D and E, and fig. S3, A and B). We used PS9 to generate VLPs encoding GFP and found that they induced GFP expression in receiver cells (Fig. 2F). These data suggest that PS9 (nt 20080-21171) is a cis-acting element that is sufficient for triggering RNA packaging into SC2-VLPs although it is not currently known whether this sequence is required for the packaging of the SARS-CoV-2 genome.

SC2-VLPs provide a new and more physiological model compared to pseudoviruses for testing mutations in all viral structural proteins (S, E, M, N) for effects on assembly, packaging and cell entry. Surprisingly, none of the 15 SC2-VLPs generated with S mutant genes, including four with the combined mutations found in the Alpha, Beta, Gamma and Epsilon variants, increased luciferase expression in transduced cells beyond that observed for the original SC2-VLPs (Fig. 3, A and B, and table S2). Since nearly all circulating variants contain the S:D614G mutation, we compared all mutants to the ancestral S protein modified to include G614 (termed WT+D614G). Minor changes in S expression between mutants may be a confounding factor since SC2-VLPs mediate luciferase expression optimally in a narrow range of S expression. Over a range of 6.25 ng to 50 pg per well of S-encoding plasmid, none of the tested S mutations produced >2-fold improvement in luciferase expression (fig. S4, A and B); slightly improved luciferase expression occurred with the S sequence derived from the Alpha variant (B.1.1.7) and S containing the mutation N501Y within the receptor binding domain. These findings contrast with prior results using S-pseudotyped

lentiviruses, where enhanced entry was reported for some S mutations including S:N501Y (*25*, *26*). However, S mutations tested in the context of SARS-CoV-2 infectious clones have shown mixed effects, and mutations within S may also mediate enhanced transmission by interfering with the binding of neutralizing antibodies (*27*, *28*). We tested whether VLPs could also be used to measure antibody neutralization and found results similar to previously reported data using a pseudovirus neutralization assay (Fig. 3C and fig. S4C). Using a neutralizing monoclonal antibody (MM43), we observed dose-dependent inhibition of luminescence with a measured IC50 of 0.35 μg/mL, similar to the manufacturer reported IC50 of 1.41 μg/mL. We also tested S from circulating variants and observed robust MM43-mediated neutralization of SC2-VLPs generated using Alpha, Beta, Gamma, Delta and Epsilon variant S proteins (Fig. 3D) and consistent with previous studies (*29*). These results show that SC2-VLPs employ S mediated entry and can be used for screening S-mutations for entry and neutralization. Although we did not observe enhanced entry due to the S mutations we tested, these mutations could still provide a fitness advantage for SARS-CoV-2 by limiting antibody mediated neutralization. Detailed characterization of S mutations and their sensitivity to neutralizing antibodies has been examined by other studies (*30*, *31*).

We next tested whether N mutations found in circulating variants improve viral particle assembly, RNA delivery and/or reporter gene expression using SC2-VLPs. We tested 15 N mutations including two combinations corresponding to the Alpha and Gamma variants since they both contain the co-occurring R203K/G204R mutations. The Alpha and Gamma variant N improved luciferase expression in receiver cells by 7.5- and 4.2-fold respectively relative to the ancestral Wuhan Hu-1 N-protein (Fig. 3E). In addition, four single amino acid changes improved luciferase expression: P199L, S202R, R203K and R203M. Two of these mutations do not change the overall charge (P199L, R203K), one results in a more positive charge (S202R), and one results in a more negative charge (R203M), suggesting that the improvement in luciferase expression is not likely due to simple electrostatics. Western blotting revealed no correlation between N protein expression levels and luciferase induction, suggesting that these N mutations enhance luciferase induction through a different mechanism (fig. S5). Interestingly, half of the amino acid changes observed within N and all of the mutations observed to enhance luminescence occur within a seven amino acid region (aa199-205) of the central disordered region (termed the "linker" region, Fig. 3F) suggestive of a shared mechanism.

Further analysis of six N mutants was conducted to determine whether these mutations affect SC2-VLP assembly efficiency, RNA packaging, or RNA uncoating prior to expression. We chose the three mutants that demonstrated ~10-fold improved luciferase expression (P199L, S202R, R203M) and two mutants that did not increase luciferase expression significantly (G204R, M234I) in the preliminary screen and compared these to the wild type (Fig. 4A). N protein expression levels were similar in packaging cells, except for G204R, and did not correlate with luciferase expression (Fig. 4, A and B). We then purified SC2-VLPs containing each N mutation and found that those containing P199L and S202R had increased levels of S, N and luciferase RNA while R203M showed increased luciferase RNA only (Fig. 4C). These results suggest that mutations within the N linker domain improve the assembly of SC2-VLPs, leading either to greater overall VLP production, a larger fraction of VLPs that contain RNA or higher RNA content per particle. In either case, these results suggest a previously unanticipated explanation for the increased fitness and spread of SARS-CoV-2 variants of concern.

To validate whether the effects we observed in SC2-VLPs improve replication of intact virus, we used reverse genetics to generate SARS-CoV-2 containing N:S202R and N:R203M substitutions within a USA/WA1-2020 (Washington isolate) background (*1*) (Fig. 4D). We generated and NGS-verified stocks of virus containing the indicated mutations (fig. S6). We infected A549-ACE2 cells with wildtype, N:S202R, or N:R203M at a multiplicity of infection (MOI) of 0.1 and collected supernatants at 24, 48 and 72 hours post infection. RT-qPCR indicated 45-fold and 23-fold higher RNA content in the supernatant at 72 hours post infection, and plaque assays indicated 166-fold and 51-fold higher infectious titers for N:S202R and N:R203M virus, respectively (Fig. 4, E to G). Our results indicate that both of these mutations enhance replication in lung epithelial cells, consistent with our observations using SC2-VLPs.

Overall, we present a strategy for rapidly generating and analyzing SC2-VLPs that package and deliver exogenous mRNA. This approach allows examination of viral assembly, budding, stability, maturation, entry and genome uncoating involving all of the viral structural proteins (S, E, M, N) without generating replication-competent virus. Such a strategy is useful not only for dissecting the molecular virology of SARS-CoV-2 but also for future development and screening of therapeutics targeting assembly, budding, maturation and entry. This strategy is ideally suited for the development of new antivirals targeting SARS-CoV-2 as it is sensitive, quantitative and scalable to high-throughput workflows. The unexpected finding of improved mRNA packaging and luciferase induction by mutations within the N protein points to a previously unknown strategy for coronaviruses to evolve enhanced viral fitness. The mechanism for this enhancement involves increased mRNA packaging and delivery although the exact process is currently unknown. Recent literature suggests that these mutations may affect phosphorylation of N,

its binding to RNA and phase separation behavior all of which could impact the efficiency of assembly (*32–34*). Our observations of enhanced RNA packaging and replication are consistent with recent reports that the Delta variant (containing N:R203M) generates 1000-fold higher levels of viral RNA within patients (*35*). Our results provide a molecular basis to explain why the SARS-CoV-2 Delta variant demonstrates improved viral fitness.

REFERENCES AND NOTES

1. X. Xie, A. Muruato, K. G. Lokugamage, K. Narayanan, X. Zhang, J. Zou, J. Liu, C. Schindewolf, N. E. Bopp, P. V. Aguilar, K. S. Plante, S. C. Weaver, S. Makino, J. W. LeDuc, V. D. Menachery, P.-Y. Shi, An Infectious cDNA Clone of SARS-CoV-2. *Cell Host Microbe* **27**, 841–848.e3 (2020). doi:10.1016/j.chom.2020.04.004 Medline
2. S. Torii, C. Ono, R. Suzuki, Y. Morioka, I. Anzai, Y. Fauzyah, Y. Maeda, W. Kamitani, T. Fukuhara, Y. Matsuura, Establishment of a reverse genetics system for SARS-CoV-2 using circular polymerase extension reaction. *Cell Rep.* **35**, 109014 (2021). doi:10.1016/j.celrep.2021.109014 Medline
3. C. Ye, K. Chiem, J.-G. Park, F. Oladunni, R. N. Platt 2nd, T. Anderson, F. Almazan, J. C. de la Torre, L. Martinez-Sobrido, Rescue of SARS-CoV-2 from a Single Bacterial Artificial Chromosome. *mBio* **11**, e02168-20 (2020). doi:10.1128/mBio.02168-20 Medline
4. X. Xie, K. G. Lokugamage, X. Zhang, M. N. Vu, A. E. Muruato, V. D. Menachery, P.-Y. Shi, Engineering SARS-CoV-2 using a reverse genetic system. *Nat. Protoc.* **16**, 1761–1784 (2021). doi:10.1038/s41596-021-00491-8 Medline
5. S. J. Rihn, A. Merits, S. Bakshi, M. L. Turnbull, A. Wickenhagen, A. J. T. Alexander, C. Baillie, B. Brennan, F. Brown, K. Brunker, S. R. Bryden, K. A. Burness, S. Carmichael, S. J. Cole, V. M. Cowton, P. Davies, C. Davis, G. De Lorenzo, C. L. Donald, M. Dorward, J. I. Dunlop, M. Elliott, M. Fares, A. da Silva Filipe, J. R. Freitas, W. Furnon, R. J. Gestuveo, A. Geyer, D. Giesel, D. M. Goldfarb, N. Goodman, R. Gunson, C. J. Hastie, V. Herder, J. Hughes, C. Johnson, N. Johnson, A. Kohl, K. Kerr, H. Leech, L. S. Lello, K. Li, G. Lieber, X. Liu, R. Lingala, C. Loney, D. Mair, M. J. McElwee, S. McFarlane, J. Nichols, K. Nomikou, A. Orr, R. J. Orton, M. Palmarini, Y. A. Parr, R. M. Pinto, S. Raggett, E. Reid, D. L. Robertson, J. Royle, N. Cameron-Ruiz, J. G. Shepherd, K. Smollett, D. G. Stewart, M. Stewart, E. Sugrue, A. M. Szemiel, A. Taggart, E. C. Thomson, L. Tong, L. S. Torrie, R. Toth, M. Varjak, S. Wang, S. G. Wilkinson, P. G. Wyatt, E. Zusinaite, D. R. Alessi, A. H. Patel, A. Zaid, S. J. Wilson, S. Mahalingam, A plasmid DNA-launched SARS-CoV-2 reverse genetics system and coronavirus toolkit for COVID-19 research. *PLOS Biol.* **19**, e3001091 (2021). doi:10.1371/journal.pbio.3001091 Medline
6. J. A. Plante, Y. Liu, J. Liu, H. Xia, B. A. Johnson, K. G. Lokugamage, X. Zhang, A. E. Muruato, J. Zou, C. R. Fontes-Garfias, D. Mirchandani, D. Scharton, J. P. Bilello, Z. Ku, Z. An, B. Kalveram, A. N. Freiberg, V. D. Menachery, X. Xie, K. S. Plante, S. C. Weaver, P.-Y. Shi, Spike mutation D614G alters SARS-CoV-2 fitness. *Nature* **592**, 116–121 (2021). doi:10.1038/s41586-020-2895-3 Medline
7. K. H. D. Crawford, R. Eguia, A. S. Dingens, A. N. Loes, K. D. Malone, C. R. Wolf, H. Y. Chu, M. A. Tortorici, D. Veesler, M. Murphy, D. Pettie, N. P. King, A. B. Balazs, J. D. Bloom, Protocol and Reagents for Pseudotyping Lentiviral Particles with SARS-CoV-2 Spike Protein for Neutralization Assays. *Viruses* **12**, 513 (2020). doi:10.3390/v12050513 Medline
8. A. A. Latif, J. L. Mullen, M. Alkuzweny, G. Tsueng, M. Cano, E. Haag, J. Zhou, M. Zeller, E. Hufbauer, N. Matteson, C. Wu, K. G. Andersen, A. I. Su, K. Gangavarapu, L. D. Hughes; the Center for Viral Systems Biology, Lineage comparison. outbreak.info (available at https://outbreak.info/compare-lineages), accessed 22 July 2021.
9. W. Zeng, G. Liu, H. Ma, D. Zhao, Y. Yang, M. Liu, A. Mohammed, C. Zhao, Y. Yang, J. Xie, C. Ding, X. Ma, J. Weng, Y. Gao, H. He, T. Jin, Biochemical characterization of SARS-CoV-2 nucleocapsid protein. *Biochem. Biophys. Res. Commun.* **527**, 618–623 (2020). doi:10.1016/j.bbrc.2020.04.136 Medline
10. J. Cubuk, J. J. Alston, J. J. Incicco, S. Singh, M. D. Stuchell-Brereton, M. D. Ward, M. I. Zimmerman, N. Vithani, D. Griffith, J. A. Wagoner, G. R. Bowman, K. B. Hall, A. Soranno, A. S. Holehouse, The SARS-CoV-2 nucleocapsid protein is dynamic, disordered, and phase separates with RNA. *Nat. Commun.* **12**, 1936 (2021). doi:10.1038/s41467-021-21953-3 Medline
11. T. M. Perdikari, A. C. Murthy, V. H. Ryan, S. Watters, M. T. Naik, N. L. Fawzi, SARS-CoV-2 nucleocapsid protein phase-separates with RNA and with human hnRNPs. *EMBO J.* **39**, e106478 (2020). doi:10.15252/embj.2020106478 Medline
12. C. B. Plescia, E. A. David, D. Patra, R. Sengupta, S. Amiar, Y. Su, R. V. Stahelin, SARS-CoV-2 viral budding and entry can be modeled using BSL-2 level virus-like particles. *J. Biol. Chem.* **296**, 100103 (2021). doi:10.1074/jbc.RA120.016148 Medline
13. H. Swann, A. Sharma, B. Preece, A. Peterson, C. Eldredge, D. M. Belnap, M. Vershinin, S. Saffarian, Minimal system for assembly of SARS-CoV-2 virus like particles. *Sci. Rep.* **10**, 21877 (2020). doi:10.1038/s41598-020-78656-w Medline
14. J. Lu, G. Lu, S. Tan, J. Xia, H. Xiong, X. Yu, Q. Qi, X. Yu, L. Li, H. Yu, N. Xia, T. Zhang, Y. Xu, J. Lin, A COVID-19 mRNA vaccine encoding SARS-CoV-2 virus-like particles induces a strong antiviral-like immune response in mice. *Cell Res.* **30**, 936–939 (2020). doi:10.1038/s41422-020-00392-7 Medline
15. Y. L. Siu, K. T. Teoh, J. Lo, C. M. Chan, F. Kien, N. Escriou, S. W. Tsao, J. M. Nicholls, R. Altmeyer, J. S. M. Peiris, R. Bruzzone, B. Nal, The M, E, and N structural proteins of the severe acute respiratory syndrome coronavirus are required for efficient assembly, trafficking, and release of virus-like particles. *J. Virol.* **82**, 11318–11330 (2008). doi:10.1128/JVI.01052-08 Medline
16. P.-K. Hsieh, S. C. Chang, C.-C. Huang, T.-T. Lee, C.-W. Hsiao, Y.-H. Kou, I.-Y. Chen, C.-K. Chang, T.-H. Huang, M.-F. Chang, Assembly of severe acute respiratory syndrome coronavirus RNA packaging signal into virus-like particles is nucleocapsid dependent. *J. Virol.* **79**, 13848–13855 (2005). doi:10.1128/JVI.79.22.13848-13855.2005 Medline
17. S. Dent, B. W. Neuman, Purification of coronavirus virions for Cryo-EM and proteomic analysis. *Coronaviruses* **1282**, 99–108 (2015). doi:10.1007/978-1-4939-2438-7_10 Medline
18. X. Lu, Y. Chen, B. Bai, H. Hu, L. Tao, J. Yang, J. Chen, Z. Chen, Z. Hu, H. Wang, Immune responses against severe acute respiratory syndrome coronavirus induced by virus-like particles in mice. *Immunology* **122**, 496–502 (2007). doi:10.1111/j.1365-2567.2007.02676.x Medline
19. L. Kuo, P. S. Masters, Functional analysis of the murine coronavirus genomic RNA packaging signal. *J. Virol.* **87**, 5182–5192 (2013). doi:10.1128/JVI.00100-13 Medline
20. K. Woo, M. Joo, K. Narayanan, K. H. Kim, S. Makino, Murine coronavirus packaging signal confers packaging to nonviral RNA. *J. Virol.* **71**, 824–827 (1997). doi:10.1128/jvi.71.1.824-827.1997 Medline
21. J. A. Fosmire, K. Hwang, S. Makino, Identification and characterization of a coronavirus packaging signal. *J. Virol.* **66**, 3522–3530 (1992). doi:10.1128/jvi.66.6.3522-3530.1992 Medline
22. A. Kanakan, N. Mishra, J. Srinivasa Vasudevan, S. Sahni, A. Khan, S. Sharma, R. Pandey, Threading the Pieces Together: Integrative Perspective on SARS-CoV-2. *Pathogens* **9**, 912 (2020). doi:10.3390/pathogens9110912 Medline
23. T. Giroglou, J. Cinatl Jr., H. Rabenau, C. Drosten, H. Schwalbe, H. W. Doerr, D. von Laer, Retroviral vectors pseudotyped with severe acute respiratory syndrome coronavirus S protein. *J. Virol.* **78**, 9007–9015 (2004). doi:10.1128/JVI.78.17.9007-9015.2004 Medline
24. M. Ujike, C. Huang, K. Shirato, S. Makino, F. Taguchi, The contribution of the cytoplasmic retrieval signal of severe acute respiratory syndrome coronavirus to intracellular accumulation of S proteins and incorporation of S protein into virus-like particles. *J. Gen. Virol.* **97**, 1853–1864 (2016). doi:10.1099/jgv.0.000494 Medline
25. X. Deng, M. A. Garcia-Knight, M. M. Khalid, V. Servellita, C. Wang, M. K. Morris, A. Sotomayor-González, D. R. Glasner, K. R. Reyes, A. S. Gliwa, N. P. Reddy, C. Sanchez San Martin, S. Federman, J. Cheng, J. Balcerek, J. Taylor, J. A. Streithorst, S. Miller, B. Sreekumar, P.-Y. Chen, U. Schulze-Gahmen, T. Y. Taha, J. M. Hayashi, C. R. Simoneau, G. R. Kumar, S. McMahon, P. V. Lidsky, Y. Xiao, P. Hemarajata, N. M. Green, A. Espinosa, C. Kath, M. Haw, J. Bell, J. K. Hacker, C. Hanson, D. A. Wadford, C. Anaya, D. Ferguson, P. A. Frankino, H. Shivram, L. F. Lareau, S. K. Wyman, M. Ott, R. Andino, C. Y. Chiu, Transmission, infectivity, and neutralization of a spike L452R SARS-CoV-2 variant. *Cell* **184**, 3426–3437.e8 (2021). doi:10.1016/j.cell.2021.04.025 Medline
26. A. Kuzmina, Y. Khalaila, O. Voloshin, A. Keren-Naus, L. Boehm-Cohen, Y. Raviv, Y. Shemer-Avni, E. Rosenberg, R. Taube, SARS-CoV-2 spike variants exhibit differential infectivity and neutralization resistance to convalescent or post-

vaccination sera. *Cell Host Microbe* **29**, 522–528.e2 (2021). doi:10.1016/j.chom.2021.03.008 Medline
27. Y. Liu, J. Liu, K. S. Plante, J. A. Plante, X. Xie, X. Zhang, Z. Ku, Z. An, D. Scharton, C. Schindewolf, V. D. Menachery, P.-Y. Shi, S. C. Weaver, The N501Y spike substitution enhances SARS-CoV-2 transmission. bioRxiv (2021), doi:10.1101/2021.03.08.434499.
28. C. Motozono, M. Toyoda, J. Zahradnik, A. Saito, H. Nasser, T. S. Tan, I. Ngare, I. Kimura, K. Uriu, Y. Kosugi, Y. Yue, R. Shimizu, J. Ito, S. Torii, A. Yonekawa, N. Shimono, Y. Nagasaki, R. Minami, T. Toya, N. Sekiya, T. Fukuhara, Y. Matsuura, G. Schreiber, T. Ikeda, S. Nakagawa, T. Ueno, K. Sato; Genotype to Phenotype Japan (G2P-Japan) Consortium, SARS-CoV-2 spike L452R variant evades cellular immunity and increases infectivity. *Cell Host Microbe* **29**, 1124–1136.e11 (2021). doi:10.1016/j.chom.2021.06.006 Medline
29. R. Kalkeri, Z. Cai, S. Lin, J. Farmer, Y. V. Kuzmichev, F. Koide, SARS-CoV-2 Spike Pseudoviruses: A Useful Tool to Study Virus Entry and Address Emerging Neutralization Escape Phenotypes. *Microorganisms* **9**, 1744 (2021). doi:10.3390/microorganisms9081744 Medline
30. Y. Weisblum, F. Schmidt, F. Zhang, J. DaSilva, D. Poston, J. C. Lorenzi, F. Muecksch, M. Rutkowska, H.-H. Hoffmann, E. Michailidis, C. Gaebler, M. Agudelo, A. Cho, Z. Wang, A. Gazumyan, M. Cipolla, L. Luchsinger, C. D. Hillyer, M. Caskey, D. F. Robbiani, C. M. Rice, M. C. Nussenzweig, T. Hatziioannou, P. D. Bieniasz, Escape from neutralizing antibodies by SARS-CoV-2 spike protein variants. *eLife* **9**, e61312 (2020). doi:10.7554/eLife.61312 Medline
31. W. T. Harvey, A. M. Carabelli, B. Jackson, R. K. Gupta, E. C. Thomson, E. M. Harrison, C. Ludden, R. Reeve, A. Rambaut, S. J. Peacock, D. L. Robertson; COVID-19 Genomics UK (COG-UK) Consortium, SARS-CoV-2 variants, spike mutations and immune escape. *Nat. Rev. Microbiol.* **19**, 409–424 (2021). doi:10.1038/s41579-021-00573-0 Medline
32. X. Ju, Y. Zhu, Y. Wang, J. Li, J. Zhang, M. Gong, W. Ren, S. Li, J. Zhong, L. Zhang, Q. C. Zhang, R. Zhang, Q. Ding, A novel cell culture system modeling the SARS-CoV-2 life cycle. *PLOS Pathog.* **17**, e1009439 (2021). doi:10.1371/journal.ppat.1009439 Medline
33. C. R. Carlson, J. B. Asfaha, C. M. Ghent, C. J. Howard, N. Hartooni, M. Safari, A. D. Frankel, D. O. Morgan, Phosphoregulation of Phase Separation by the SARS-CoV-2 N Protein Suggests a Biophysical Basis for its Dual Functions. *Mol. Cell* **80**, 1092–1103.e4 (2020). doi:10.1016/j.molcel.2020.11.025 Medline
34. S. Lu, Q. Ye, D. Singh, Y. Cao, J. K. Diedrich, J. R. Yates 3rd, E. Villa, D. W. Cleveland, K. D. Corbett, The SARS-CoV-2 nucleocapsid phosphoprotein forms mutually exclusive condensates with RNA and the membrane-associated M protein. *Nat. Commun.* **12**, 502 (2021). Medline
35. B. Li, A. Deng, K. Li, Y. Hu, Z. Li, Q. Xiong, Z. Liu, Q. Guo, L. Zou, H. Zhang, M. Zhang, F. Ouyang, J. Su, W. Su, J. Xu, H. Lin, J. Sun, J. Peng, H. Jiang, P. Zhou, T. Hu, M. Luo, Y. Zhang, H. Zheng, J. Xiao, T. Liu, R. Che, H. Zeng, Z. Zheng, Y. Huang, J. Yu, L. Yi, J. Wu, J. Chen, H. Zhong, X. Deng, M. Kang, O. G. Pybus, M. Hall, K. A. Lythgoe, Y. Li, J. Yuan, J. He, J. Lu, Viral infection and Transmission in a large well-traced outbreak caused by the Delta SARS-CoV-2 variant, medRxiv (2021); doi:10.1101/2021.07.07.21260122.

ACKNOWLEDGMENTS

We thank Dr. Pei-Yong Shi for providing the plasmids for SARS-CoV-2 reverse genetics. We would also like to acknowledge Dr. Jennifer Hamilton for advice and guidance and Dr. Stacia Wyman and Bryan Bach for sequencing of infectious clones. We thank Satish Pillai and Hannah S. Sperbert at Vitalant Research Institute (San Francisco, CA) for providing the 293T-ACE2-TMPRSS2 cells used for pseudovirus entry assays. We thank Sean P.J. Whelan lab for providing Vero cells overexpressing human TMPRSS2. We also thank Olivier Schwartz from Institut Pasteur for providing A549 cells stably expressing ACE2 (A549-ACE2). We thank Karl-Klaus Conzelmann for providing BSR-T7/5 cells. **Funding:** This project was funded by a grant from the National Institutes of Health (R21AI59666) and by support from the Howard Hughes Medical Institute and the Gladstone Institutes. AMS acknowledges the support of the Natural Sciences and Engineering Research Council of Canada (NSERC PDF-533021-2019) and IPC support from the NIH (F31 AI164671-01). **Author contributions:** Conceptualization: AMS, JAD. Investigation: AMS, TYT, TT, IPC, AC, MMK, BS, P-YC, JMH, KMS. Methodology: AMS, TYT, TT, JAD, MO. Supervision: JAD, MO. Writing: AMS, TYT, TT, JAD. **Competing interests:** AMS and JAD are inventors on a patent application filed by the Gladstone Institutes and the University of California that covers the method and composition of SARS-CoV-2 VLP preparations for RNA transduction and expression in cells. **Data and materials availability:** All data are available in the main paper or supplementary material. Plasmids are available from Addgene (addgene.org) or by request. This work is licensed under a Creative Commons Attribution 4.0 International (CC BY 4.0) license, which permits unrestricted use, distribution, and reproduction in any medium, provided the original work is properly cited. To view a copy of this license, visit https://creativecommons.org/licenses/by/4.0/. This license does not apply to figures/photos/artwork or other content included in the article that is credited to a third party; obtain authorization from the rights holder before using such material.

SUPPLEMENTARY MATERIALS

science.org/doi/10.1126/science.abl6184
Materials and Methods
Figs. S1 to S6
Tables S1 to S3
References
MDAR Reproducibility Checklist

26 July 2021; accepted 29 October 2021
Published online 4 November 2021
10.1126/science.abl6184

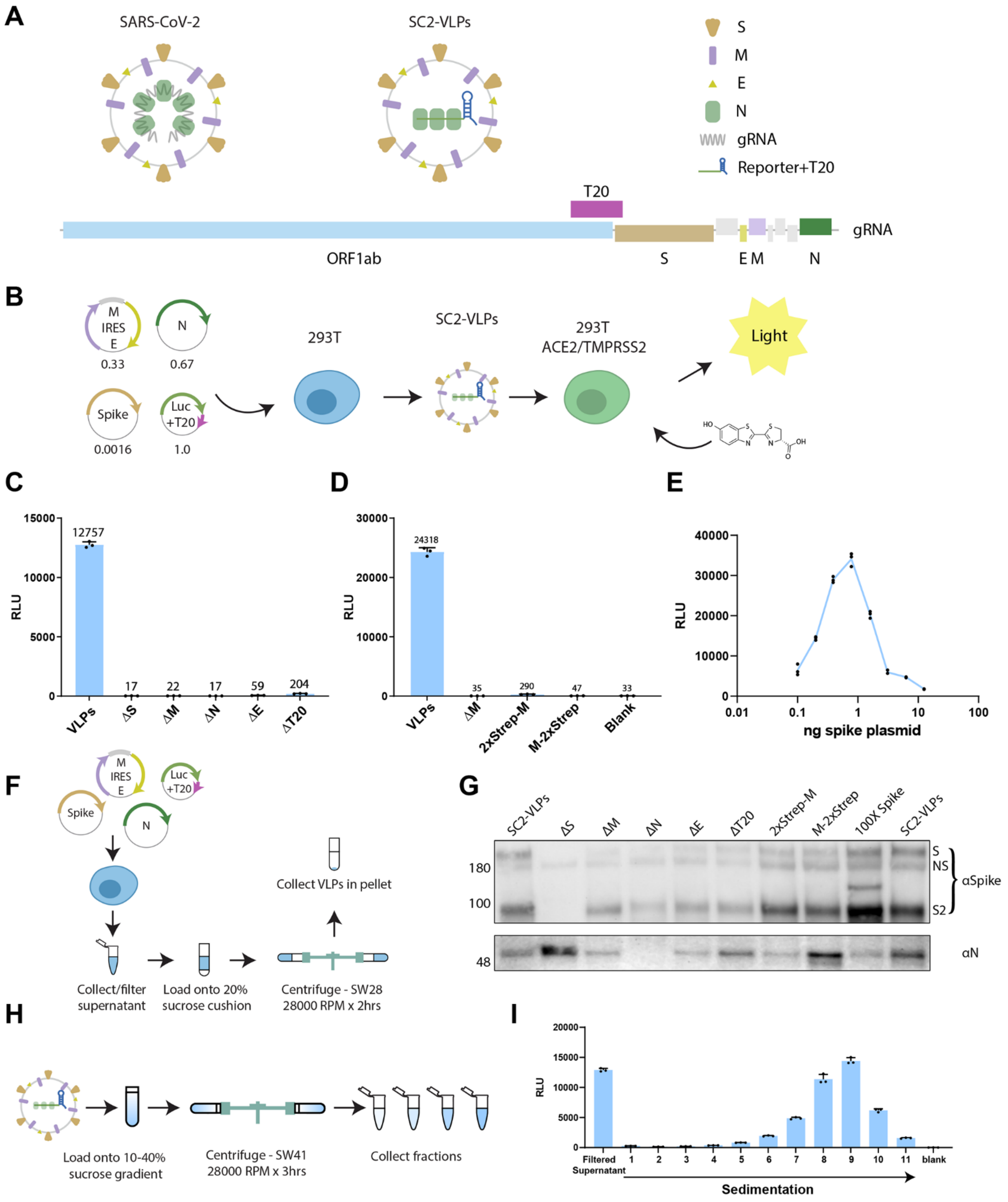
A
SARS-CoV-2
SC2-VLPs
S
M
E
N
gRNA
Reporter+T20
T20
ORF1ab
S
E M
N
gRNA
B
M
IRES
E
0.33
N
0.67
Spike
0.0016
Luc
+T20
1.0
293T
SC2-VLPs
293T
ACE2/TMPRSS2
Light
C
RLU
12757
17
22
17
59
204
VLPs
ΔS
ΔM
ΔN
ΔE
ΔT20
D
RLU
24318
35
290
47
33
VLPs
ΔM
2xStrep-M
M-2xStrep
Blank
E
RLU
ng spike plasmid
F
Collect VLPs in pellet
Collect/filter supernatant
Load onto 20% sucrose cushion
Centrifuge - SW28 28000 RPM x 2hrs
G
SC2-VLPs
ΔS
ΔM
ΔN
ΔE
ΔT20
2xStrep-M
M-2xStrep
100X Spike
SC2-VLPs
180
100
48
S
NS
S2
αSpike
αN
H
Load onto 10-40% sucrose gradient
Centrifuge - SW41 28000 RPM x 3hrs
Collect fractions
I
RLU
Filtered Supernatant
blank
Sedimentation

Fig. 1 (previous page). Design and characterization of SC2-VLPs. (**A**) Schematic of SARS-CoV-2 and SC2-VLP design and location of RNA packaging sequence T20. (**B**) Process for generating and detecting luciferase encoding SC2-VLPs. Numbers below plasmid maps indicate ratios used for transfection. (**C**) Induced luciferase expression measured in receiver cells (293T-ACE2/TMPRSS2) from “Standard” SC2-VLPs containing S, M, N, E and luciferase-T20 transcript as well as VLPs lacking each one of the components. (**D**) N- or C-terminal 2x strep-tag on M abrogates vector induced luciferase expression. (**E**) Optimal luciferase expression requires a narrow range of spike plasmid concentrations corresponding to 1/500th of the total plasmid mass used for transfection. (**F**) Schematic for purification of SC2-VLPs. (**G**) Western blot showing S and N in pellets purified from standard SC2-VLPs and conditions that did not induce luciferase expression in receiver cells. (**H**) Schematic for sucrose gradient for separating SC2-VLPs. (**I**) Induced luciferase expression from sucrose gradient fractions of SC2-VLPs. S2: Cleavage product of S. NS: Non-specific band. Error bars indicate standard deviation with N = 3 independent transfections in each case.

Fig. 2 (next page). RNA packaging into SC2-VLPs by SARS-CoV-2 sequences. (**A**) Arrayed screen for determining the location of the optimal sequence for RNA packaging in SC2-VLPs. 2-kb tiled segments of the genome were cloned into the 3′ UTR of the luciferase plasmid. (**B**) Induced luciferase expression in receiver cells by SC2-VLPs containing different tiled segments from the SARS-CoV-2 genome. (**C**) Heatmap visualization of the data from (B) showing the locations of tiled segments relative to the SARS-CoV-2 genome. Color intensity indicates luminescence of receiver cells for each tile normalized to expression for luciferase plasmid containing no insert. (**D**) Smaller segments of the genome were used to locate the optimal RNA packaging sequence. (**E**) Heatmap visualization of the data from (D). (**F**) Flow cytometry analysis of GFP expression in 293T ACE2/TMPRSS2 cells incubated with SC2-VLPs encoding GFP-PS9, GFP (no packaging sequence) or no VLPs. Error bars indicate standard deviation with N = 3 independent transfections in each case.

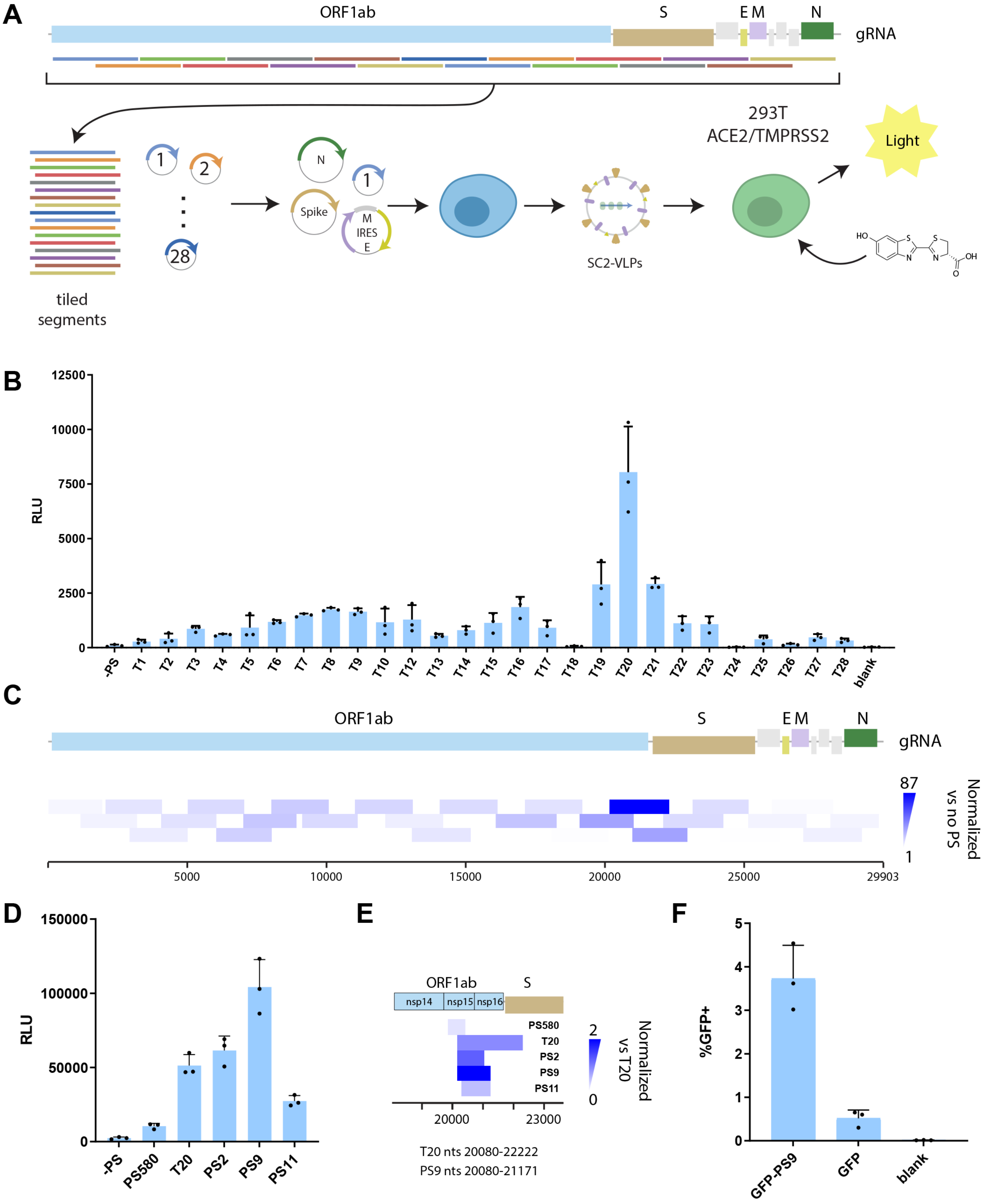
A
ORF1ab
S
E M
N
gRNA
tiled segments
1
2
28
N
Spike
M IRES E
293T ACE2/TMPRSS2
Light
SC2-VLPs
B
RLU
12500
10000
7500
5000
2500
0
-PS T1 T2 T3 T4 T5 T6 T7 T8 T9 T10 T12 T13 T14 T15 T16 T17 T18 T19 T20 T21 T22 T23 T24 T25 T26 T27 T28 blank
C
ORF1ab
S
E M
N
gRNA
87
1
Normalized vs no PS
5000
10000
15000
20000
25000
29903
D
RLU
150000
100000
50000
0
-PS PS580 T20 PS2 PS9 PS11
E
ORF1ab
S
nsp14 nsp15 nsp16
PS580
T20
PS2
PS9
PS11
2
0
Normalized vs T20
20000
23000
T20 nts 20080-22222
PS9 nts 20080-21171
F
%GFP+
5
4
3
2
1
0
GFP-PS9 GFP blank

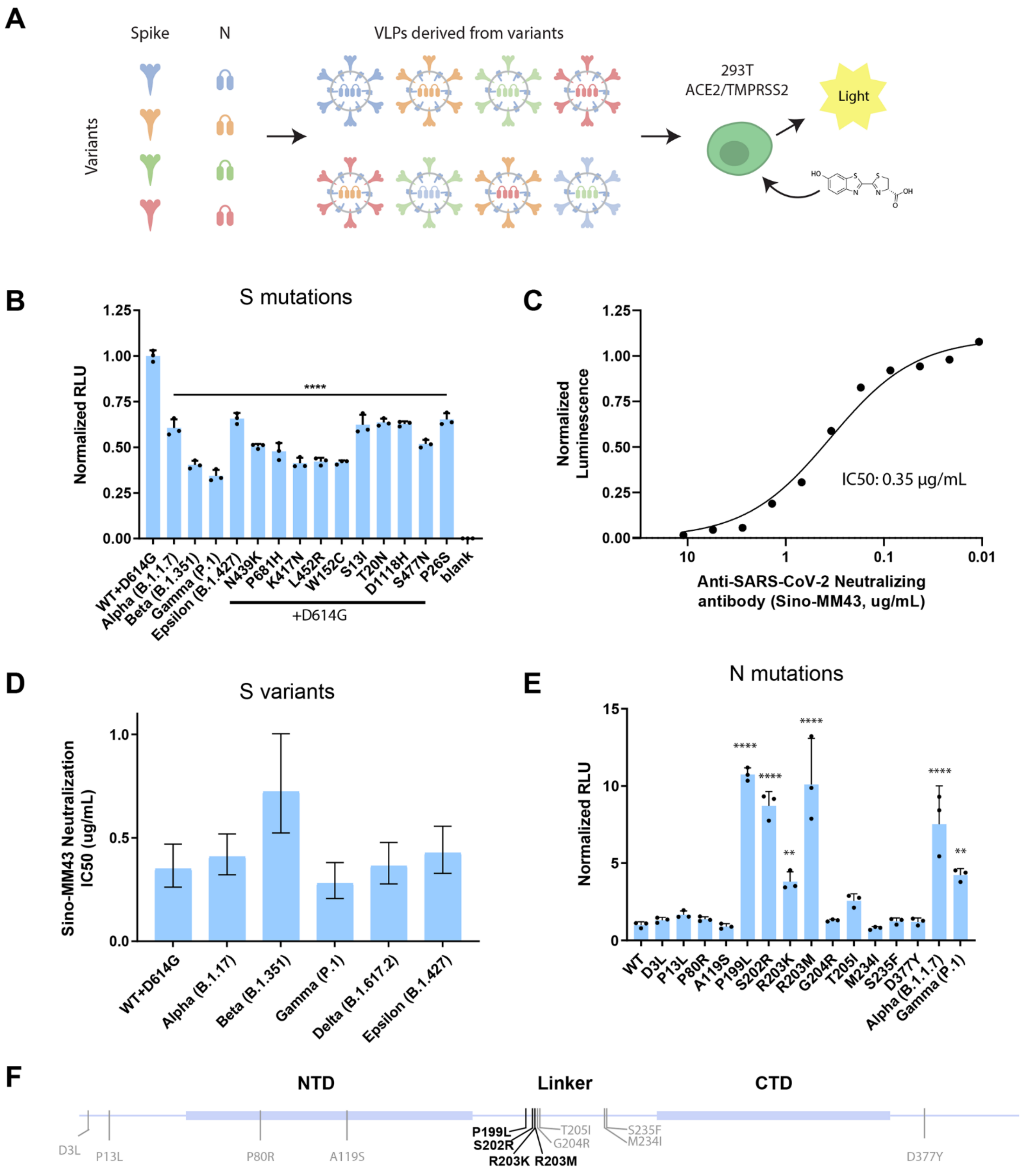
A
Spike
N
VLPs derived from variants
Variants
293T
ACE2/TMPRSS2
Light
B
S mutations
Normalized RLU
****
WT+D614G
Alpha (B.1.1.7)
Beta (B.1.351)
Gamma (P.1)
Epsilon (B.1.427)
N439K
P681H
K417N
L452R
W152C
S13I
T20N
D1118H
S477N
P26S
blank
+D614G
C
Normalized Luminescence
IC50: 0.35 µg/mL
Anti-SARS-CoV-2 Neutralizing antibody (Sino-MM43, ug/mL)
D
S variants
Sino-MM43 Neutralization IC50 (ug/mL)
WT+D614G
Alpha (B.1.17)
Beta (B.1.351)
Gamma (P.1)
Delta (B.1.617.2)
Epsilon (B.1.427)
E
N mutations
Normalized RLU
WT
D3L
P13L
P80R
A119S
P199L
S202R
R203K
R203M
G204R
T205I
M234I
S235F
D377Y
Alpha (B.1.1.7)
Gamma (P.1)
F
NTD
Linker
CTD
D3L
P13L
P80R
A119S
P199L
S202R
R203K
R203M
T205I
G204R
S235F
M234I
D377Y

Fig. 3 (previous page). Effect of mutations in the S and N proteins on SC2-VLP induced luminescence. (**A**) Schematic for cloning and testing mutations observed in SARS-CoV-2 variants using SC2-VLPs. (**B**) Initial screen of 15 S mutants compared to a reference ancestral S containing the D614G mutation (termed WT). Details of mutations listed in table S2. (**C**) Neutralization curve for SC2-VLPs generated using ancestral S and neutralized with anti-S antibody MM43 (SinoBiological, cat# 40591). (**D**) Neutralization IC50 of S variants using SC2-VLPs and MM43. (**E**) Initial screen of 15 N mutants compared to the reference Wuhan Hu-1 N sequence (WT). Details of mutations listed in table S3. (**F**) Map of SARS-CoV-2 N domains showing the locations of observed mutations. Mutations observed to enhance signal are bolded. (B and E) Error bars indicate standard deviation with N = 3 independent transfections in each case. Significance was determined by one-way analysis of variance and multiple comparisons using Holm-Šídák test. $^{**}p < 0.01$, $^{****}p < 0.0001$. (E) Error bars indicate 95% confidence intervals derived from curve fitting in Graphpad Prism.

Fig. 4 (next page). Impact of mutations in SARS-CoV-2 N on RNA packaging and viral titer. (**A**) Luciferase expression in receiver cells from six N mutants re-tested after preparation in a larger batch. (**B**) Relative N-expression of selected mutants in packaging cells normalized to WT using GAPDH as a loading control. (**C**) Western blot (protein) and Northern blot (RNA) of VLPs generated using N-mutants purified by ultracentrifugation. Lentivirus was added before ultracentrifugation to allow utilization of p24 as an internal control. (**D**) Schematic for reverse genetics system used to generate mutant SARS-CoV-2. (**E**) RT-qPCR of supernatant collected from A549-ACE2 cells infected with WT and mutant SARS-CoV-2 at MOI of 0.1 at 24, 48, and 72 hours post infection. (**F**) Representative plaques and (**G**) quantification of infectious viral titers from the same experiment. MOI: Multiplicity of infection; CTD: C-terminal domain. Error bars indicate standard deviation with N = 3 independent transfections/infections in each case. Significance was determined by one-way analysis of variance and multiple comparisons using Holm-Šídák test. $^{**}p < 0.01$, $^{****}p < 0.0001$.

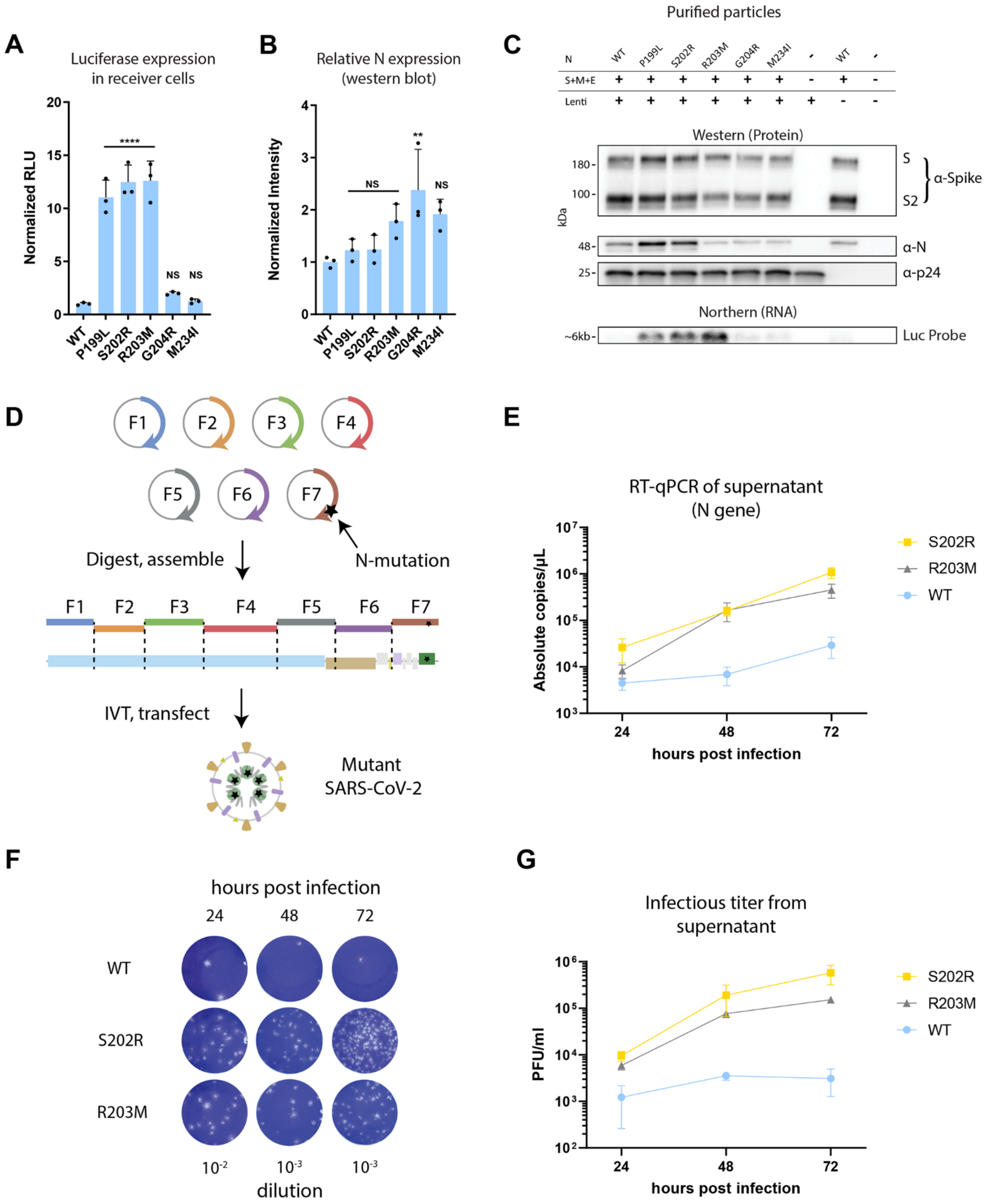

A
Luciferase expression in receiver cells
Normalized RLU
****
NS
NS
WT
P199L
S202R
R203M
G204R
M234I
B
Relative N expression (western blot)
Normalized Intensity
NS
**
NS
C
Purified particles
N
S+M+E
Lenti
Western (Protein)
kDa
180
100
48
25
S
S2
α-Spike
α-N
α-p24
Northern (RNA)
~6kb
Luc Probe
D
F1
F2
F3
F4
F5
F6
F7
Digest, assemble
N-mutation
IVT, transfect
Mutant SARS-CoV-2
E
RT-qPCR of supernatant (N gene)
Absolute copies/µL
hours post infection
S202R
R203M
WT
F
hours post infection
24
48
72
WT
S202R
R203M
dilution
G
Infectious titer from supernatant
PFU/ml
hours post infection
