# Supplementary material for: Rapid assessment of SARS-CoV-2–evolved variants using virus-like particles
Source: Science. 2021 Nov 4;374(6575):1626–32. doi: 10.1126/science.abl6184 (PMC9005165; doi:10.1126/science.abl6184)
Supplement: Supplementary file 2 — Materials and Methods Figs. S1 to S6 Tables S1 to S3 References [file science.abl6184_sm.pdf]

Science
AAAS

Supplementary Materials for

Rapid assessment of SARS-CoV-2–evolved variants using virus-like particles

Abdullah M. Syed *et al.*

Corresponding authors: Melanie Ott, melanie.ott@gladstone.ucsf.edu; Jennifer A. Doudna, doudna@berkeley.edu

Science **374**, 1626 (2021)
DOI: 10.1126/science.abl6184

The PDF file includes:

Materials and Methods
Figs. S1 to S6
Tables S1 to S3
References

Other Supplementary Material for this manuscript includes the following:

MDAR Reproducibility Checklist

Materials and methods

Cloning for plasmids encoding structural proteins: pcDNA3.1 backbone plasmids were generated encoding N, and M-IRES-E. Sequences for E, M and N were PCR amplified from codon optimized plasmids were gifts from Nevan Krogan (Addgene plasmid # 141385, 141386, 141391,). pcDNA3.1-SARS2-Spike was a gift from Fang Li (Addgene plasmid # 145032). Site directed mutagenesis (NEB) was used to remove the C9-tag and introduce the D614G mutation.

Cloning of SARS-CoV-2 genome tiled segments: RNA was extracted from SARS-CoV-2 (Washington isolate) viral supernatant inactivated in Trizol by phase separation. RNA was reverse transcribed using protoscript II (NEB) and tiled segments (T1-T28) were PCR amplified from cDNA using primers compatible with ligation independent cloning (LIC). Tiles were cloned into a plasmid containing luciferase with a LIC destination site in the 3'UTR.

SC2-VLP production: For a 6-well, plasmids Cov2-N (0.67), CoV2-M-IRES-E (0.33), CoV-2-Spike (0.0016) and Luc-T20 (1.0) at indicated mass ratios for a total of 4 µg of DNA were diluted in 200 µL optimem. 12 µg PEI was diluted in 200 µL Opti-MEM and added to plasmid dilution quickly to complex the DNA. Transfection mixture was incubated for 20 minutes at room temperature and then added dropwise to 293T cells in 2 mL of DMEM containing fetal bovine serum and penicillin/streptomycin. Media was changed after 24 hours of transfection and At 48 hours post-transfection, VLP containing supernatant was collected and filtered using a 0.45 µm syringe filter. For other culture sizes, the mass of DNA used was 1 µg for 24-well, 4 µg for 6-well, 20 µg for 10-cm plate and 60 µg for 15-cm plate. Optimem volumes were 100 µL, 400 µL, 1 mL and 3 mL respectively and PEI was always used at 3:1 mass ratio.

Luciferase readout: In each well of a clear 96-well plate 50 µL of SC2-VLP containing supernatant was added to 50 µL of cell suspension containing 30 000 receiver cells (293T

ACE2/TMPRSS2). Cells were allowed to attach and take up VLPs overnight. Next day, supernatant was removed and cells were rinsed with 1X PBS and lysed in 20 µL passive lysis buffer (Promega) for 15 minutes at room temperature with gentle rocking. Lysates were transferred to an opaque white 96-well plate and 50 µL of reconstituted luciferase assay buffer was added and mixed with each lysate. Luminescence was measured immediately after mixing using a TECAN plate reader.

SC2-VLP purification using sucrose cushion: SC2-VLP produced in 10-cm plates (10 mL of culture) were added to 13.2 mL ultracentrifuge tubes. 1 mL of 20% sucrose was underlaid using a 4" blunt needle. VLPs were centrifuged for 2 hours at 28 000 RPM using a SW41 Ti swinging bucket rotor. Supernatant was removed and ultracentrifuge tubes were inverted for 5 minutes on a paper towel with gentle tapping to remove remaining supernatant. VLPs were resuspended in 50 µL phosphate buffered saline for further experiments.

SC2-VLP PEG precipitation: 0.136 volumes of polyethylene glycol stock (50% PEG, 2.2% NaCl) was added to filtered supernatants containing SC2-VLPs to achieve a final concentration of 6% PEG. Solution was mixed thoroughly and precipitation was allowed to proceed for 2hrs at 4°C and then centrifuged at 2 000g for 20 minutes. Supernatant was discarded and VLPs were resuspended in PBS.

SC2-VLP concentration using Amicon filters: 0.5 mL filtered supernatant was added to 0.5 mL 100 kDa molecular weight cutoff amicon filters and centrifuged for 30 minutes at 2 000g. Concentrate was diluted in 1X PBS containing 0.02% Tween 20 for all wash steps.

Western blot: For western blots of lysates, media was removed and cells were rinsed with PBS. Cells were then lysed for 20 minutes in RIPA lysis buffer containing Halt protease and phosphatase inhibitor cocktail. For western blots of ultracentrifuge concentrated VLPs, 10 mL of

VLP supernatant from a 10-cm plate was pelleted (28 000 RPM, 2hrs, SW41 Ti, 1mL 20% sucrose cushion), the supernatant was discarded and VLPs were resuspended in 50 μL of PBS. 15 μL of concentrated VLPs were used to western blot. Laemmli loading buffer (1x final) and dithiothreitol (DTT, 40 mM final) was added to lysates or VLP solution and heated for 95°C for 5 minutes to lyse VLPs and denature proteins. Samples were loaded on to 12-40% gradient gels (Biorad) and transferred to a PVDF membrane (Biorad). Membrane was blocked in 10% NFDM and stained with primary antibody: anti-N (abcam ab273434, 1:500 dilution), anti-S (abcam ab272504, 1:1000), anti-GAPDH (Santa Cruz sc-365062, 1:1000), anti-p24 (Sigma, 1:2000) for 2 hours at room temperature. Blots were rinsed with TBS-T three times for 10 minutes each and stained with secondary (abcam ab205719 (mouse), 1:5000). Imaged using pierce chemiluminescence kit.

Sucrose gradient fractionation: 10% to 40% sucrose gradient was prepared using a gradient mixer in 13.2 mL ultracentrifuge tubes. Concentrated and resuspended SC2-VLPs were overlaid on top of the gradient and centrifuged in a SW41 Ti rotor for 3 hours at 28 000 RPM. Gradient was fractionated from the bottom using a 4" blunt needle and a peristaltic pump. For cell infection, each fraction was diluted 20X and added to 293T cells expressing ACE2/TMPRSS2. Luciferase signal was measured the next day.

GFP-VLPs and flow cytometry. GFP was cloned into the luciferase destination vector (Luc-no PS) and Luc-PS9 to generate GFP-LIC and GFP-PS9. VLPs were generated in 10-cm plates and concentrated through a 20% sucrose cushion. 50 μL of concentrated VLPs were added to each well of a 24-well plate along with 120 000 receiver cells (293T ACE2/TMPRSS2). Cells were incubated with VLPs overnight and GFP expression was measured the next day using flow cytometry.

Northern Blot: VLPs collected from a 10-cm plate were concentrated by ultracentrifugation through a 20% sucrose cushion (28 000 RPM, 2hrs, SW41 Ti). The supernatant was discarded and VLPs were resuspended in 50 µL of PBS. 20 µL of concentrated VLPs were used for Northern blotting. VLPs were lysed by adding 500 µL of Trizol (Sigma) and RNA was extracted by phase separation, precipitated with isopropanol with GlycoBlue and washed with 75% ethanol. RNA was resuspended in 30 µL of water, added to 30 µL 2x RNA Loading Dye (NEB) and denatured at 65°C for 15 minutes then loaded onto a 1% agarose gel containing 1X MOPS and 4% formaldehyde. Samples were run at room temperature for 12hrs at 20V and transferred by capillary action to Nylon membrane. The membrane was hybridized with a ^{32}P-labeled luciferase DNA probe (Promega) and visualized using a phosphoscreen on a Typhoon imager (GE).

Construction of SARS-CoV2 mutant viruses: Seven cDNA fragments spanning the USA/WA1-2020 SARS-CoV-2 genome were a kind gift from Dr. Pei-Yong Shi. SARS-CoV2 nucleocapsid S202R and R203M mutant viruses were constructed by mutagenesis of a previously described seven-fragment cDNA clone USA/WA1-2020 SARS-CoV-2 (*4*). Briefly, N:S202R and N:R203M mutations were introduced into fragment 7 (F7) (pCC1-CoV2-F7) using site-directed mutagenesis. To assemble the full length SARS-CoV2 cDNA, cDNA fragments were obtained by restriction enzyme digestion (BsaI for F1-4 and Esp3I/PvuI for F5-6 and Esp3I/SnaBI for F7), and purification by gel extraction (Qiagen gel extraction kit). In vitro ligation of the fragments was done in three steps: 1) ligation of F1-3, F4-5, and F6-7 separately; 2) ligation of F1-3 and F4-5 to obtain F1-5; 3) ligation of F1-5 to F6-7 to obtain F1-7. All ligations were performed with T4 DNA ligase overnight at 4°C. The full length F1-7 cDNA was phenol/chloroform extracted, isopropanol precipitated, and resuspended in nuclease-free water. SARS-CoV2 full length gRNA

was produced by using mMESSAGE mMACHINE T7 Transcription Kit (ThermoFisher Scientific). The purified SARS-CoV2 full length wild type and mutant gRNAs were electroporated into BSR-T7/T5 cells, which are baby hamster kidney-21 (BHK-21) cells constitutively expressing T7 RNA polymerase and co-cultured with Vero-TMPRSS2 cells along with WT N RNA. After 3-4 days or until CPE is observed, mutant viruses (P0) were collected and used to infect Vero-TMPRSS2 cells to produce P1 viral stocks that were utilized for subsequent infection experiments. All viral sequences were verified by NGS (fig. S6). Viral titers were determined by plaque assays on Vero-TMPRSS2 cells. Virus preparation and experiments were performed in the BSL3 facility.

Cell lines: Cells were maintained in a humidified incubator at 37°C in 5% CO_2 in the indicated media and passaged every 3-4 days. 293T cells were obtained from ATCC and maintained in DMEM with 10% FBS and 1% penicillin/streptomycin. 293T-ACE2/TMPRSS2 were a gift from Satish Pillai lab and cultured in DMEM with 10% FBS, 1% P/S, 2 µg/ml puromycin and 10 µg/ml blasticidin. Vero E6 was cultured in DMEM supplemented with 10% FBS and 2mM glutamine. Vero cells overexpressing human TMPRSS2, a kind gift from the Whelan lab (Case et al., 2020), were grown in DMEM with 10% FBS and 1x glutamine. A549 cells stably expressing ACE2 (A549-ACE2) were a gift from O. Schwartz. A549-ACE2 cells were cultured in DMEM supplemented with 10% FBS, 2mM glutamine and blasticidin (20 µg/ml) (Sigma). Short Terminal Repeat (STR) analysis by the Berkeley Cell Culture Facility on 17 July 2020 authenticates these as A549 cells with 100% probability.

Infection and titration of infectious clones: A549-ACE2 were inoculated with WT, S202R, or R203M mutant viruses at multiplicity of infection (MOI) 0.1 in triplicate for 1 hr, After the infection, the cells were washed with PBS to remove the unattached virus and then cultured for

72 hours. Medium was changed and cell culture supernatants were collected daily and stored -80°C for virus titration by plaque assay. Cells and cell culture supernatants were also collected for RNA analysis.

Plaque assays: Vero-TMPRSS2 were plated 2.5 x 10^5 cells per well in 12-well plates overnight. Cell culture supernatants were serially diluted in DMEM with 10% FBS and 300 µl of diluted inoculum were added to Vero-TMPRSS2 monolayers at 37C, 5% CO_2 for 1 hr, followed by an Avicel (IMCD) overlay. After 3 days, plaques were fixed with 10% formalin and visualized by staining with crystal violet.

Supplementary Figure 1

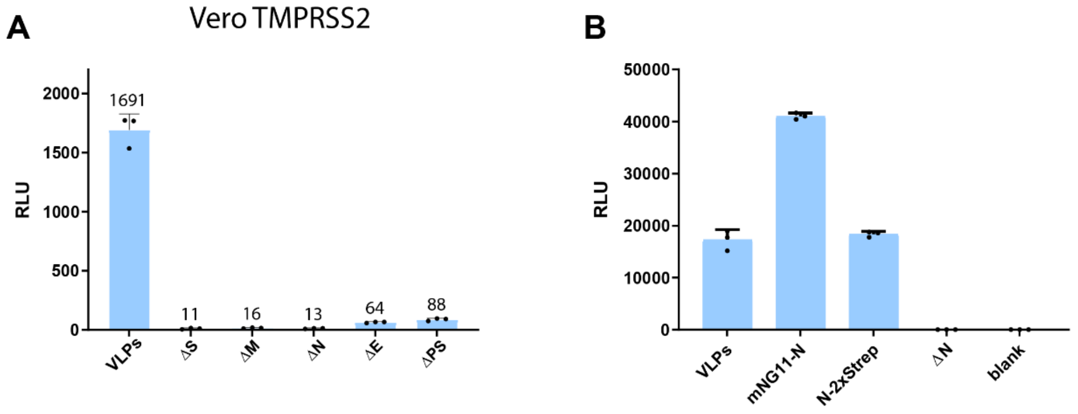

Fig. S1. Requirements for induced expression by SC2-VLPs. A) Luminescence measured from Vero E6 cells incubated with standard SC2-VLPs as well as missing either S, M, N, E or the packaging signal (PS). B) Luminescence from receiver cells after incubation with standard SC2-VLPs as well as tagged N (mNG11-N: N with amino-terminal mNG11 tag and N-2xStrep: N with carboxy-terminal 2xStrep tag). Error bars indicate standard deviation with N=3 independent transfections in each case.

Supplementary Figure 2

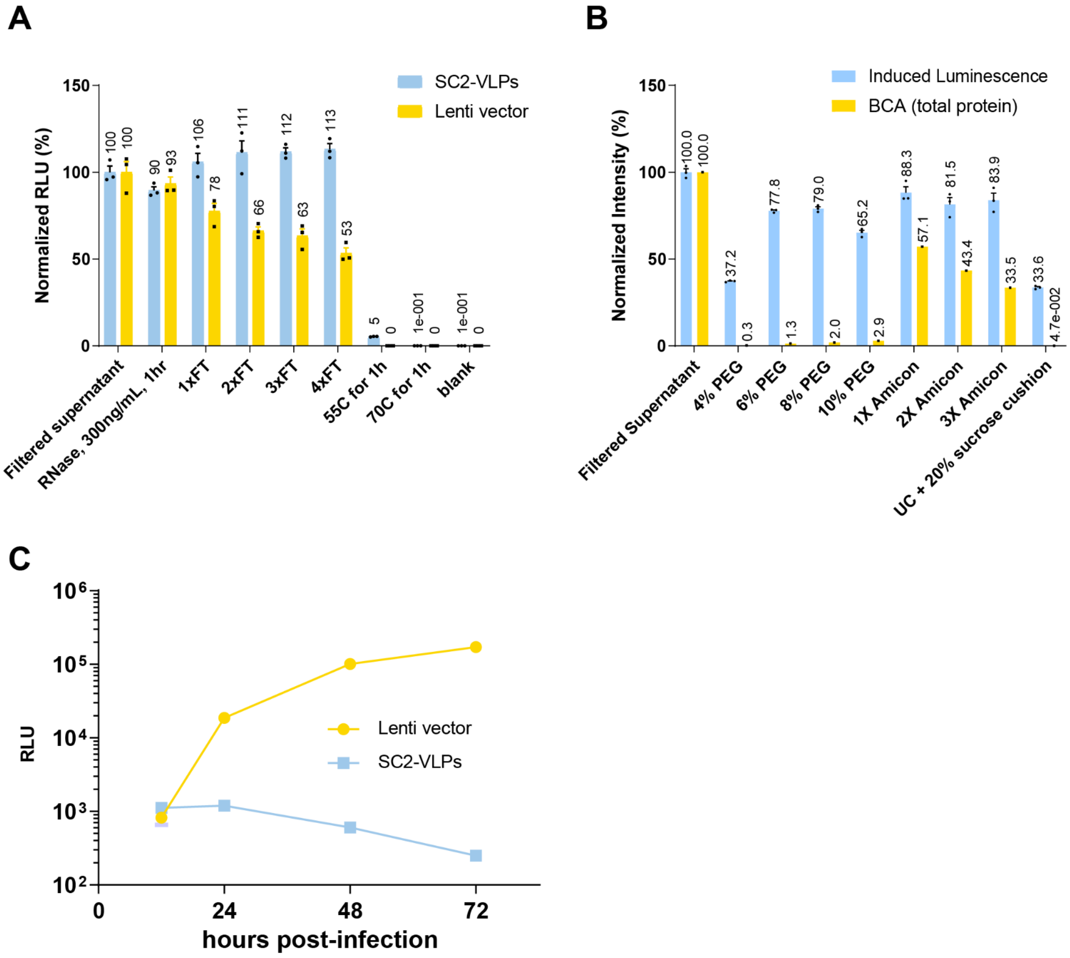

Fig. S2. Characterization of SC2-VLPs stability and purification. A) Luminescence induced in receiver cells from SC2-VLPs after treatment with ribonuclease A or 1-4 freeze-thaw cycles or incubation at 55°C and 70°C, respectively. All values were normalized to the original supernatant. Lentiviral particles encoding luciferase are shown as comparison. B) Induced luminescence from SC2-VLPs purified/concentrated using different methods compared to total protein measurement from the same samples using bicinchoninic acid (BCA) assay. C) Luminescence measured from 293T-AC2/TMPRSS2 receiver cells at 12, 24, 48, and 72 hours after incubation with either S-pseudotyped lentivirus or SC2-VLPs encoding luciferase. Error bars indicate standard deviation with N=3 independent transfections in each case.

Supplementary Figure 3

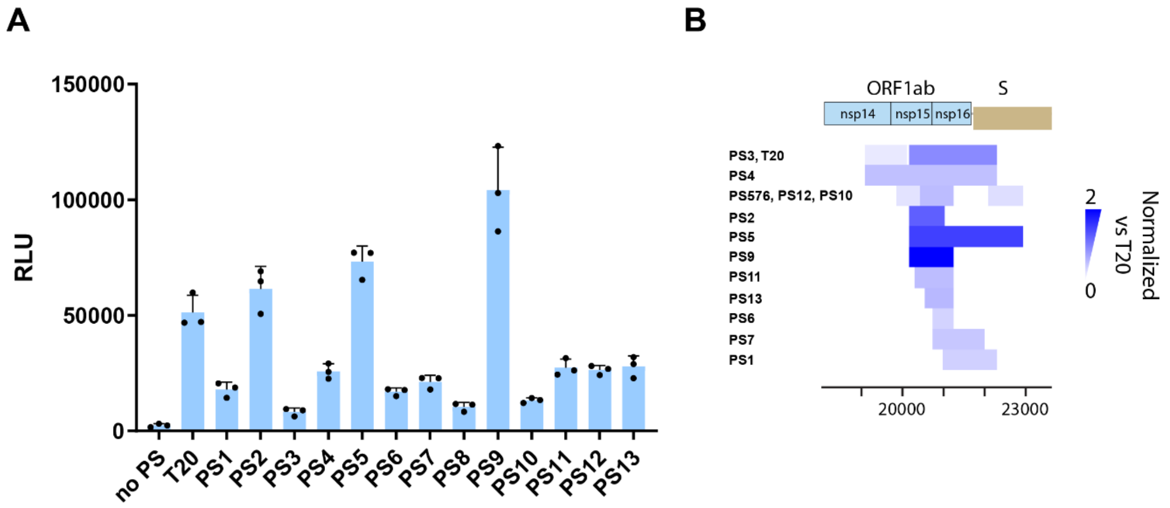

Fig. S3. Minimal sequence required for specific packaging into SC2-VLPs. A) Induced luminescence in receiver cells after incubation with SC2-VLPs containing a transcript expressing luciferase. The luciferase transcript contains varying segments from SARS-CoV-2 genome shown graphically in (B). “no PS” indicates luciferase only transcript. Color in (B) indicates the observed luminescence normalized to the T20 transcript. Error bars indicate standard deviation with N=3 independent transfections/infections in each case.

Supplementary Figure 4

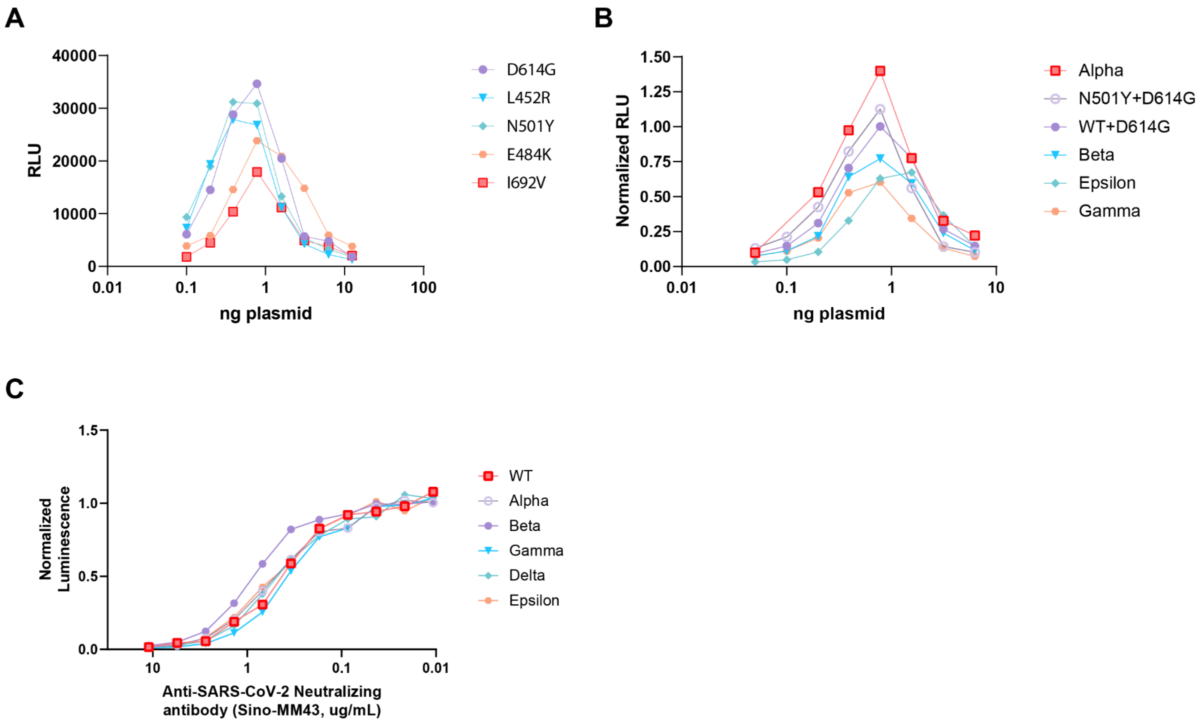

Fig. S4. Effect of spike mutations on SC2-VLPs and neutralization. A, B) Induced luminescence from receiver cells incubated with SC2-VLPs containing varying concentrations of and mutations within the S plasmid. S plasmid ranging from 0.1 ng to 12.5 ng was added to each well of a 24-well plate. Total DNA used for transfection (N, M-IRES-E, T20) was 1 µg for each well. C) Neutralization curves of S variants against antibody MM43 (SinoBiological). IC50 from each shown in Fig. 3D.

Supplementary Figure 5

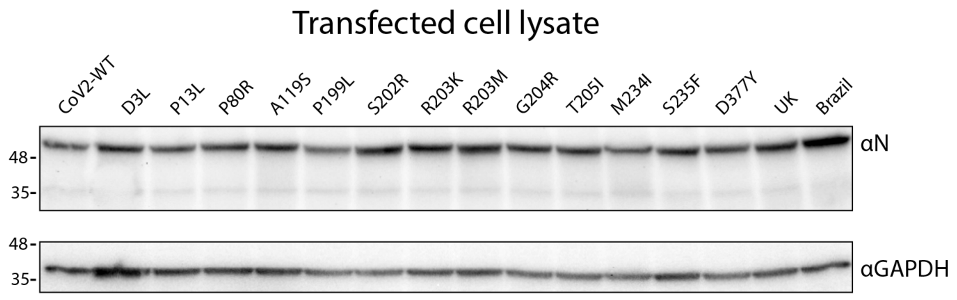

Fig. S5. Expression levels of N mutants. Western blot of lysates from packaging cells transfected with N mutations stained using anti-N antibody (top) and anti-GAPDH antibody (bottom). Expression levels are similar between mutants and do not correlate with induced luminescence from SC2-VLPs made from these mutants.

Supplementary Figure 6

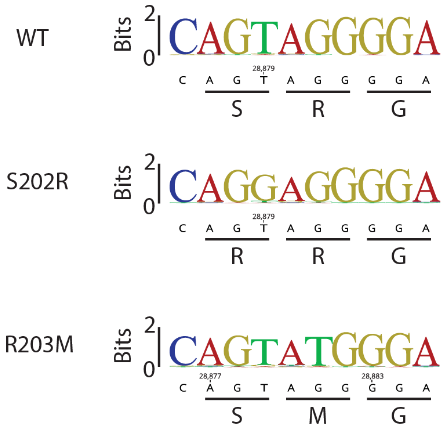

Fig. S6. Sequence logo visualization of sequencing data from infectious clones generated by reverse genetics. Arrows indicate sites that were modified.

Table S1. Nucleotide start and end positions of sequences T1-T28 and PS1-PS9 relative to the Wuhan Hu-1 reference genome.

Sequence name	Start position	End position
T1	18	1932
T2	1172	3179
T3	2070	4075
T4	2936	5010
T5	4054	6038
T6	5004	7020
T7	6018	8009
T8	6996	8898
T9	8006	10043
T10	9101	11072
T11	10013	12088
T12	11001	13055
T13	12074	14137
T14	13028	15133
T15	14010	16085
T16	16011	18186
T17	17068	19115
T18	18020	20047
T19	19018	20955
T20	20080	22222
T21	20912	22873
T22	22016	24166
T23	23050	25077
T24	24038	26169
T25	25056	27130
T26	25906	28096
T27	27029	29125
T28	27987	29727

PS1	20912	22221
PS2	20080	20954
PS3	19018	20046
PS4	19018	22221
PS5	20080	22872
PS6	20654	21171
PS7	20654	21920
PS8	19785	20348
PS9	20080	21171
PS10	22016	22872
PS11	20231	21171
PS12	20352	21171
PS13	20468	21171

Table S2. CoV-2 S mutations and variants screened relative to Wuhan Hu-1 reference genome.

Label	Mutations
WT+D614G	D614G
Alpha (B.1.1.7)	del69,70, del144, D614G, N501Y, A570D, P681H, T716I, S982A, D1118H
Beta (B.1.351)	K417N, E484K, N501Y, D614G
Gamma (P.1)	L13F, T20N, P26S, D138Y, R190S,K417T,E484K,N501Y,D614G,H655Y,T1027I, V1176F
Epsilon (B.1.427)	S13I, W152C, L452R, D614G
N439K	N439K, D614G
P681H	D614G, P681H
K417N	K417N, D614G
L452R	L452R, D614G
W152C	W152C, D614G
S13I	S13I, D614G
T20N	T20N, D614G
D1118H	D614G, D1118H

S477N	S477N, D614G
P26S	P26S, D614G

Table S3. CoV-2 N mutations and variants screened relative to Wuhan Hu-1 reference genome.

Label	Mutations
WT	
D3L	D3L
P13L	P13L
P80R	P80R
A119S	A119S
P199L	P199L
S202R	S202R
R203K	R203K
R203M	R203M
G204R	G204R
T205I	T205I
M234I	M234I
S235F	S235F
D377Y	D377Y
Alpha	D3L, R203K, G204R, S235F
Gamma (P.1)	P80R, R203K, G204R

References and Notes

1. X. Xie, A. Muruato, K. G. Lokugamage, K. Narayanan, X. Zhang, J. Zou, J. Liu, C. Schindewolf, N. E. Bopp, P. V. Aguilar, K. S. Plante, S. C. Weaver, S. Makino, J. W. LeDuc, V. D. Menachery, P.-Y. Shi, An Infectious cDNA Clone of SARS-CoV-2. *Cell Host Microbe* **27**, 841–848.e3 (2020). doi:10.1016/j.chom.2020.04.004 Medline

2. S. Torii, C. Ono, R. Suzuki, Y. Morioka, I. Anzai, Y. Fauzyah, Y. Maeda, W. Kamitani, T. Fukuhara, Y. Matsuura, Establishment of a reverse genetics system for SARS-CoV-2 using circular polymerase extension reaction. *Cell Rep.* **35**, 109014 (2021). doi:10.1016/j.celrep.2021.109014 Medline

3. C. Ye, K. Chiem, J.-G. Park, F. Oladunni, R. N. Platt 2nd, T. Anderson, F. Almazan, J. C. de la Torre, L. Martinez-Sobrido, Rescue of SARS-CoV-2 from a Single Bacterial Artificial Chromosome. *mBio* **11**, e02168-20 (2020). doi:10.1128/mBio.02168-20 Medline

4. X. Xie, K. G. Lokugamage, X. Zhang, M. N. Vu, A. E. Muruato, V. D. Menachery, P.-Y. Shi, Engineering SARS-CoV-2 using a reverse genetic system. *Nat. Protoc.* **16**, 1761–1784 (2021). doi:10.1038/s41596-021-00491-8 Medline

5. S. J. Rihn, A. Merits, S. Bakshi, M. L. Turnbull, A. Wickenhagen, A. J. T. Alexander, C. Baillie, B. Brennan, F. Brown, K. Brunker, S. R. Bryden, K. A. Burness, S. Carmichael, S. J. Cole, V. M. Cowton, P. Davies, C. Davis, G. De Lorenzo, C. L. Donald, M. Dorward, J. I. Dunlop, M. Elliott, M. Fares, A. da Silva Filipe, J. R. Freitas, W. Furnon, R. J. Gestuveo, A. Geyer, D. Giesel, D. M. Goldfarb, N. Goodman, R. Gunson, C. J. Hastie, V. Herder, J. Hughes, C. Johnson, N. Johnson, A. Kohl, K. Kerr, H. Leech, L. S. Lello, K. Li, G. Lieber, X. Liu, R. Lingala, C. Loney, D. Mair, M. J. McElwee, S. McFarlane, J. Nichols, K. Nomikou, A. Orr, R. J. Orton, M. Palmarini, Y. A. Parr, R. M. Pinto, S. Raggett, E. Reid, D. L. Robertson, J. Royle, N. Cameron-Ruiz, J. G. Shepherd, K. Smollett, D. G. Stewart, M. Stewart, E. Sugrue, A. M. Szemiel, A. Taggart, E. C. Thomson, L. Tong, L. S. Torrie, R. Toth, M. Varjak, S. Wang, S. G. Wilkinson, P. G. Wyatt, E. Zusinaite, D. R. Alessi, A. H. Patel, A. Zaid, S. J. Wilson, S. Mahalingam, A plasmid DNA-launched SARS-CoV-2 reverse genetics system and coronavirus toolkit for COVID-19 research. *PLOS Biol.* **19**, e3001091 (2021). doi:10.1371/journal.pbio.3001091 Medline

6. J. A. Plante, Y. Liu, J. Liu, H. Xia, B. A. Johnson, K. G. Lokugamage, X. Zhang, A. E. Muruato, J. Zou, C. R. Fontes-Garfias, D. Mirchandani, D. Scharton, J. P. Bilello, Z. Ku, Z. An, B. Kalveram, A. N. Freiberg, V. D. Menachery, X. Xie, K. S. Plante, S. C. Weaver, P.-Y. Shi, Spike mutation D614G alters SARS-CoV-2 fitness. *Nature* **592**, 116–121 (2021). doi:10.1038/s41586-020-2895-3 Medline

7. K. H. D. Crawford, R. Eguia, A. S. Dingens, A. N. Loes, K. D. Malone, C. R. Wolf, H. Y. Chu, M. A. Tortorici, D. Veesler, M. Murphy, D. Pettie, N. P. King, A. B. Balazs, J. D. Bloom, Protocol and Reagents for Pseudotyping Lentiviral Particles with SARS-CoV-2 Spike Protein for Neutralization Assays. *Viruses* **12**, 513 (2020). doi:10.3390/v12050513 Medline

8. A. A. Latif, J. L. Mullen, M. Alkuzweny, G. Tsueng, M. Cano, E. Haag, J. Zhou, M. Zeller, E. Hufbauer, N. Matteson, C. Wu, K. G. Andersen, A. I. Su, K. Gangavarapu, L. D. Hughes;

the Center for Viral Systems Biology, Lineage comparison. outbreak.info (accessed 22 July 2021); https://outbreak.info/compare-lineages.

9. W. Zeng, G. Liu, H. Ma, D. Zhao, Y. Yang, M. Liu, A. Mohammed, C. Zhao, Y. Yang, J. Xie, C. Ding, X. Ma, J. Weng, Y. Gao, H. He, T. Jin, Biochemical characterization of SARS-CoV-2 nucleocapsid protein. *Biochem. Biophys. Res. Commun.* **527**, 618–623 (2020). doi:10.1016/j.bbrc.2020.04.136 Medline

10. J. Cubuk, J. J. Alston, J. J. Incicco, S. Singh, M. D. Stuchell-Brereton, M. D. Ward, M. I. Zimmerman, N. Vithani, D. Griffith, J. A. Wagoner, G. R. Bowman, K. B. Hall, A. Soranno, A. S. Holehouse, The SARS-CoV-2 nucleocapsid protein is dynamic, disordered, and phase separates with RNA. *Nat. Commun.* **12**, 1936 (2021). doi:10.1038/s41467-021-21953-3 Medline

11. T. M. Perdikari, A. C. Murthy, V. H. Ryan, S. Watters, M. T. Naik, N. L. Fawzi, SARS-CoV-2 nucleocapsid protein phase-separates with RNA and with human hnRNPs. *EMBO J.* **39**, e106478 (2020). doi:10.15252/embj.2020106478 Medline

12. C. B. Plescia, E. A. David, D. Patra, R. Sengupta, S. Amiar, Y. Su, R. V. Stahelin, SARS-CoV-2 viral budding and entry can be modeled using BSL-2 level virus-like particles. *J. Biol. Chem.* **296**, 100103 (2021). doi:10.1074/jbc.RA120.016148 Medline

13. H. Swann, A. Sharma, B. Preece, A. Peterson, C. Eldredge, D. M. Belnap, M. Vershinin, S. Saffarian, Minimal system for assembly of SARS-CoV-2 virus like particles. *Sci. Rep.* **10**, 21877 (2020). doi:10.1038/s41598-020-78656-w Medline

14. J. Lu, G. Lu, S. Tan, J. Xia, H. Xiong, X. Yu, Q. Qi, X. Yu, L. Li, H. Yu, N. Xia, T. Zhang, Y. Xu, J. Lin, A COVID-19 mRNA vaccine encoding SARS-CoV-2 virus-like particles induces a strong antiviral-like immune response in mice. *Cell Res.* **30**, 936–939 (2020). doi:10.1038/s41422-020-00392-7 Medline

15. Y. L. Siu, K. T. Teoh, J. Lo, C. M. Chan, F. Kien, N. Escriou, S. W. Tsao, J. M. Nicholls, R. Altmeyer, J. S. M. Peiris, R. Bruzzone, B. Nal, The M, E, and N structural proteins of the severe acute respiratory syndrome coronavirus are required for efficient assembly, trafficking, and release of virus-like particles. *J. Virol.* **82**, 11318–11330 (2008). doi:10.1128/JVI.01052-08 Medline

16. P.-K. Hsieh, S. C. Chang, C.-C. Huang, T.-T. Lee, C.-W. Hsiao, Y.-H. Kou, I.-Y. Chen, C.-K. Chang, T.-H. Huang, M.-F. Chang, Assembly of severe acute respiratory syndrome coronavirus RNA packaging signal into virus-like particles is nucleocapsid dependent. *J. Virol.* **79**, 13848–13855 (2005). doi:10.1128/JVI.79.22.13848-13855.2005 Medline

17. S. Dent, B. W. Neuman, Purification of coronavirus virions for Cryo-EM and proteomic analysis. *Coronaviruses* **1282**, 99–108 (2015). doi:10.1007/978-1-4939-2438-7_10 Medline

18. X. Lu, Y. Chen, B. Bai, H. Hu, L. Tao, J. Yang, J. Chen, Z. Chen, Z. Hu, H. Wang, Immune responses against severe acute respiratory syndrome coronavirus induced by virus-like particles in mice. *Immunology* **122**, 496–502 (2007). doi:10.1111/j.1365-2567.2007.02676.x Medline

19. L. Kuo, P. S. Masters, Functional analysis of the murine coronavirus genomic RNA packaging signal. *J. Virol.* **87**, 5182–5192 (2013). doi:10.1128/JVI.00100-13 Medline

20. K. Woo, M. Joo, K. Narayanan, K. H. Kim, S. Makino, Murine coronavirus packaging signal confers packaging to nonviral RNA. *J. Virol.* **71**, 824–827 (1997). doi:10.1128/jvi.71.1.824-827.1997 Medline

21. J. A. Fosmire, K. Hwang, S. Makino, Identification and characterization of a coronavirus packaging signal. *J. Virol.* **66**, 3522–3530 (1992). doi:10.1128/jvi.66.6.3522-3530.1992 Medline

22. A. Kanakan, N. Mishra, J. Srinivasa Vasudevan, S. Sahni, A. Khan, S. Sharma, R. Pandey, Threading the Pieces Together: Integrative Perspective on SARS-CoV-2. *Pathogens* **9**, 912 (2020). doi:10.3390/pathogens9110912 Medline

23. T. Giroglou, J. Cinatl Jr., H. Rabenau, C. Drosten, H. Schwalbe, H. W. Doerr, D. von Laer, Retroviral vectors pseudotyped with severe acute respiratory syndrome coronavirus S protein. *J. Virol.* **78**, 9007–9015 (2004). doi:10.1128/JVI.78.17.9007-9015.2004 Medline

24. M. Ujike, C. Huang, K. Shirato, S. Makino, F. Taguchi, The contribution of the cytoplasmic retrieval signal of severe acute respiratory syndrome coronavirus to intracellular accumulation of S proteins and incorporation of S protein into virus-like particles. *J. Gen. Virol.* **97**, 1853–1864 (2016). doi:10.1099/jgv.0.000494 Medline

25. X. Deng, M. A. Garcia-Knight, M. M. Khalid, V. Servellita, C. Wang, M. K. Morris, A. Sotomayor-González, D. R. Glasner, K. R. Reyes, A. S. Gliwa, N. P. Reddy, C. Sanchez San Martin, S. Federman, J. Cheng, J. Balcerek, J. Taylor, J. A. Streithorst, S. Miller, B. Sreekumar, P.-Y. Chen, U. Schulze-Gahmen, T. Y. Taha, J. M. Hayashi, C. R. Simoneau, G. R. Kumar, S. McMahon, P. V. Lidsky, Y. Xiao, P. Hemarajata, N. M. Green, A. Espinosa, C. Kath, M. Haw, J. Bell, J. K. Hacker, C. Hanson, D. A. Wadford, C. Anaya, D. Ferguson, P. A. Frankino, H. Shivram, L. F. Lareau, S. K. Wyman, M. Ott, R. Andino, C. Y. Chiu, Transmission, infectivity, and neutralization of a spike L452R SARS-CoV-2 variant. *Cell* **184**, 3426–3437.e8 (2021). doi:10.1016/j.cell.2021.04.025 Medline

26. A. Kuzmina, Y. Khalaila, O. Voloshin, A. Keren-Naus, L. Boehm-Cohen, Y. Raviv, Y. Shemer-Avni, E. Rosenberg, R. Taube, SARS-CoV-2 spike variants exhibit differential infectivity and neutralization resistance to convalescent or post-vaccination sera. *Cell Host Microbe* **29**, 522–528.e2 (2021). doi:10.1016/j.chom.2021.03.008 Medline

27. Y. Liu, J. Liu, K. S. Plante, J. A. Plante, X. Xie, X. Zhang, Z. Ku, Z. An, D. Scharton, C. Schindewolf, S. G. Widen, V. D. Menachery, P.-Y. Shi, S. C. Weaver, The N501Y spike substitution enhances SARS-CoV-2 infection and transmission. *Nature* 10.1038/s41586-021-04245-0 (2021). doi:10.1038/s41586-021-04245-0 Medline.

28. C. Motozono, M. Toyoda, J. Zahradnik, A. Saito, H. Nasser, T. S. Tan, I. Ngare, I. Kimura, K. Uriu, Y. Kosugi, Y. Yue, R. Shimizu, J. Ito, S. Torii, A. Yonekawa, N. Shimono, Y. Nagasaki, R. Minami, T. Toya, N. Sekiya, T. Fukuhara, Y. Matsuura, G. Schreiber, T. Ikeda, S. Nakagawa, T. Ueno, K. Sato; Genotype to Phenotype Japan (G2P-Japan) Consortium, SARS-CoV-2 spike L452R variant evades cellular immunity and increases infectivity. *Cell Host Microbe* **29**, 1124–1136.e11 (2021). doi:10.1016/j.chom.2021.06.006 Medline

29. R. Kalkeri, Z. Cai, S. Lin, J. Farmer, Y. V. Kuzmichev, F. Koide, SARS-CoV-2 Spike Pseudoviruses: A Useful Tool to Study Virus Entry and Address Emerging Neutralization

Escape Phenotypes. *Microorganisms* **9**, 1744 (2021). doi:10.3390/microorganisms9081744 Medline

30. Y. Weisblum, F. Schmidt, F. Zhang, J. DaSilva, D. Poston, J. C. Lorenzi, F. Muecksch, M. Rutkowska, H.-H. Hoffmann, E. Michailidis, C. Gaebler, M. Agudelo, A. Cho, Z. Wang, A. Gazumyan, M. Cipolla, L. Luchsinger, C. D. Hillyer, M. Caskey, D. F. Robbiani, C. M. Rice, M. C. Nussenzweig, T. Hatziioannou, P. D. Bieniasz, Escape from neutralizing antibodies by SARS-CoV-2 spike protein variants. *eLife* **9**, e61312 (2020). doi:10.7554/eLife.61312 Medline
31. W. T. Harvey, A. M. Carabelli, B. Jackson, R. K. Gupta, E. C. Thomson, E. M. Harrison, C. Ludden, R. Reeve, A. Rambaut, S. J. Peacock, D. L. Robertson; COVID-19 Genomics UK (COG-UK) Consortium, SARS-CoV-2 variants, spike mutations and immune escape. *Nat. Rev. Microbiol.* **19**, 409–424 (2021). doi:10.1038/s41579-021-00573-0 Medline
32. X. Ju, Y. Zhu, Y. Wang, J. Li, J. Zhang, M. Gong, W. Ren, S. Li, J. Zhong, L. Zhang, Q. C. Zhang, R. Zhang, Q. Ding, A novel cell culture system modeling the SARS-CoV-2 life cycle. *PLOS Pathog.* **17**, e1009439 (2021). doi:10.1371/journal.ppat.1009439 Medline
33. C. R. Carlson, J. B. Asfaha, C. M. Ghent, C. J. Howard, N. Hartooni, M. Safari, A. D. Frankel, D. O. Morgan, Phosphoregulation of Phase Separation by the SARS-CoV-2 N Protein Suggests a Biophysical Basis for its Dual Functions. *Mol. Cell* **80**, 1092–1103.e4 (2020). doi:10.1016/j.molcel.2020.11.025 Medline
34. S. Lu, Q. Ye, D. Singh, Y. Cao, J. K. Diedrich, J. R. Yates 3rd, E. Villa, D. W. Cleveland, K. D. Corbett, The SARS-CoV-2 nucleocapsid phosphoprotein forms mutually exclusive condensates with RNA and the membrane-associated M protein. *Nat. Commun.* **12**, 502 (2021). doi:10.1038/s41467-020-20768-y Medline
35. B. Li, A. Deng, K. Li, Y. Hu, Z. Li, Q. Xiong, Z. Liu, Q. Guo, L. Zou, H. Zhang, M. Zhang, F. Ouyang, J. Su, W. Su, J. Xu, H. Lin, J. Sun, J. Peng, H. Jiang, P. Zhou, T. Hu, M. Luo, Y. Zhang, H. Zheng, J. Xiao, T. Liu, R. Che, H. Zeng, Z. Zheng, Y. Huang, J. Yu, L. Yi, J. Wu, J. Chen, H. Zhong, X. Deng, M. Kang, O. G. Pybus, M. Hall, K. A. Lythgoe, Y. Li, J. Yuan, J. He, J. Lu, Viral infection and Transmission in a large well-traced outbreak caused by the Delta SARS-CoV-2 variant, medRxiv 2021.07.07.21260122 [Preprint] (2021); doi:10.1101/2021.07.07.21260122.
